# Mutations at hypothetical binding site 2 in insulin and insulin-like growth factors 1 and 2 result in receptor- and hormone-specific responses

**DOI:** 10.1074/jbc.RA119.010072

**Published:** 2019-09-26

**Authors:** Kateřina Macháčková, Květoslava Mlčochová, Pavlo Potalitsyn, Kateřina Hanková, Ondřej Socha, Miloš Buděšínský, Anja Muždalo, Martin Lepšík, Michaela Černeková, Jelena Radosavljević, Milan Fábry, Katarína Mitrová, Martina Chrudinová, Jingjing Lin, Yevgen Yurenko, Pavel Hobza, Irena Selicharová, Lenka Žáková, Jiří Jiráček

**Affiliations:** ‡ Institute of Organic Chemistry and Biochemistry, Czech Academy of Sciences, 166 10 Prague 6, Czech Republic; § Institute of Molecular Genetics, Czech Academy of Sciences, 166 37 Prague 6, Czech Republic

**Keywords:** insulin, insulin-like growth factor (IGF), complex, mutagenesis, structural biology, structure-function, receptor tyrosine kinase, peptide hormone, hormone analog, molecular dynamics, NMR structure, receptor autophosphorylation, receptor binding

## Abstract

Information on how insulin and insulin-like growth factors 1 and 2 (IGF-1 and -2) activate insulin receptors (IR-A and -B) and the IGF-1 receptor (IGF-1R) is crucial for understanding the difference in the biological activities of these peptide hormones. Cryo-EM studies have revealed that insulin uses its binding sites 1 and 2 to interact with IR-A and have identified several critical residues in binding site 2. However, mutagenesis studies suggest that Ile-A10, Ser-A12, Leu-A13, and Glu-A17 also belong to insulin's site 2. Here, to resolve this discrepancy, we mutated these insulin residues and the equivalent residues in IGFs. Our findings revealed that equivalent mutations in the hormones can result in differential biological effects and that these effects can be receptor-specific. We noted that the insulin positions A10 and A17 are important for its binding to IR-A and IR-B and IGF-1R and that A13 is important only for IR-A and IR-B binding. The IGF-1/IGF-2 positions 51/50 and 54/53 did not appear to play critical roles in receptor binding, but mutations at IGF-1 position 58 and IGF-2 position 57 affected the binding. We propose that IGF-1 Glu-58 interacts with IGF-1R Arg-704 and belongs to IGF-1 site 1, a finding supported by the NMR structure of the less active Asp-58–IGF-1 variant. Computational analyses indicated that the aforementioned mutations can affect internal insulin dynamics and inhibit adoption of a receptor-bound conformation, important for binding to receptor site 1. We provide a molecular model and alternative hypotheses for how the mutated insulin residues affect activity.

## Introduction

**Figure 1.**
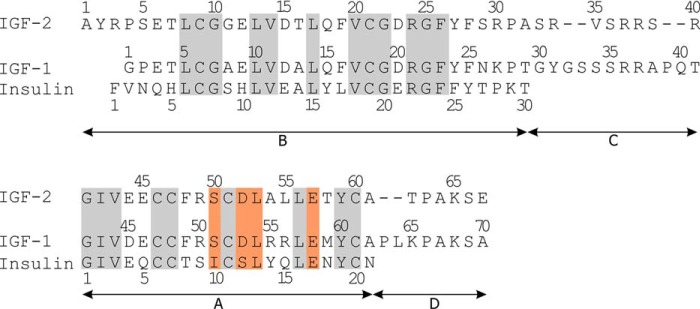
**Primary sequences of human insulin, IGF-1, and IGF-2.** The residues mutated in this study are highlighted with an *orange background*, and homologous residues are highlighted with a *gray background*.

Insulin, insulin-like growth factors 1 and 2 (IGF-1 and -2)^4^ are hormones with similar primary sequences (Fig. 1) and 3D structures and common evolutionary origins. They elicit different but partly overlapping biological functions, primarily metabolic for insulin and mainly mitogenic for both IGFs (1, 2).

These hormones trigger their functions by binding to three highly homologous (50–85% sequence homology) transmembrane glycoprotein receptors for insulin and IGF-1 (3), which belong to a large family of receptor tyrosine-kinases (4). The receptors are formed by two extracellular α-subunits and two membrane-spanning β-subunits. The receptor subunits are interconnected by several disulfide bridges to form the (αβ)_2_ heterodimer. There are two isoforms of the insulin receptor, IR-A and IR-B, which differ only by a 12-amino acid segment at the C terminus of the extracellular α-subunit (called the α-CT peptide) (5). The receptor for IGF-1 (IGF-1R) contains, similarly to IR-A, a shorter version of the α-CT peptide (3). Moreover, IR-A, IR-B, and IGF-1R can form what are known as hybrid receptors, composed of one pair of αβ subunits from one receptor and the second αβ pair from another receptor (6). The IR-A, IR-B, and IGF-1R receptors have different tissue distributions and can bind individual hormones with different affinities (7). Furthermore, the availability of IGFs is modulated by a family of six IGF-binding proteins (8), and IGF-2 also binds to a distinct receptor for IGF-2 (9, 10). All these factors contribute to the different physiological functions of the hormones. Defects in the functioning of the insulin–IGF network can have severe consequences that can result in two types of diabetes (type I and type II), growth disorders, cancer, or Alzheimer disease (11–13).

It is broadly accepted that the insulin molecule interacts with the receptor by an interplay of two binding sites: the primary binding site 1, which binds receptors with a high affinity (∼6 nm), and the secondary binding site 2, which binds receptors with a lower affinity (∼400 nm) (14, 15). Binding of insulin sites 1 and 2 to respective sites 1′ and 2′ of the receptor creates a high-affinity complex (∼0.2 nm), which induces a structural change in the extracellular domains of the receptor, transmission of the signal through the cell membrane, and activation of intracellular tyrosine-kinase subunits. Structural information is still incomplete on how the interaction of insulin and IGFs with the receptors activates intracellular signaling.

**Figure 2.**
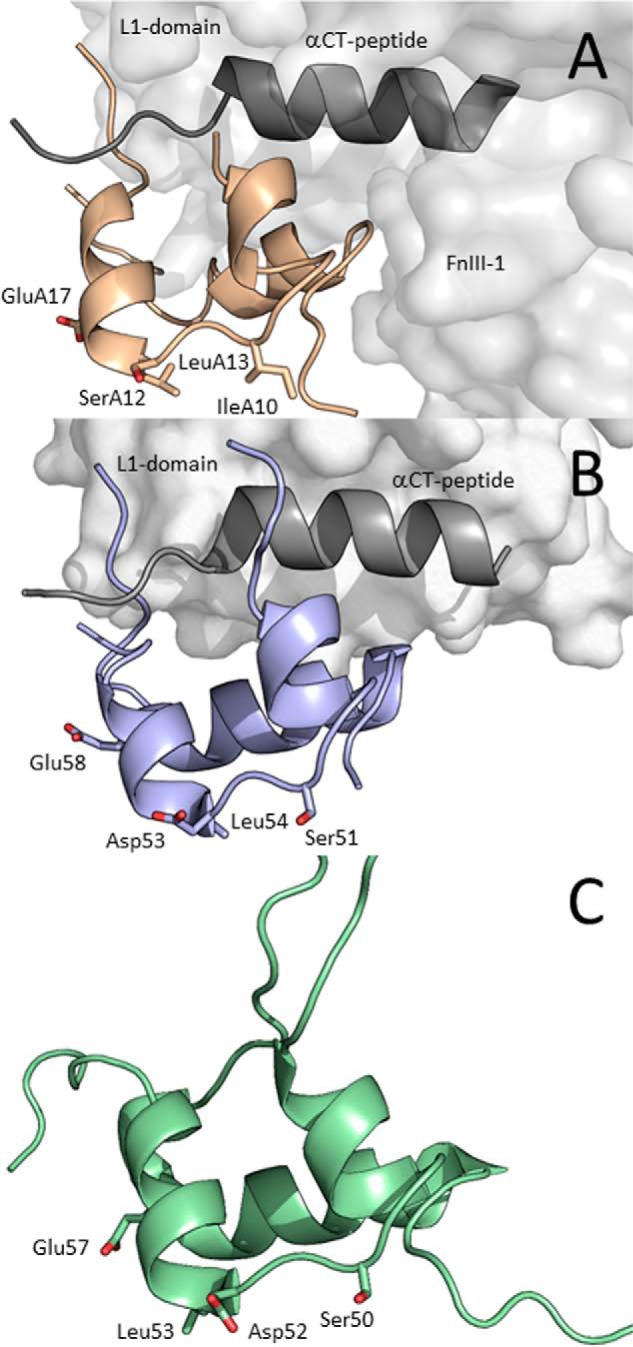
**Receptor-bound structures of insulin and IGF-1 and NMR structure of IGF-2.** *A*, cryo-EM structure of IR-A–bound insulin (PDB code 6HN5 from Ref. 22, in *light brown*). Receptor site 1′ is represented by the L1 domain (*light gray*), and αCT peptide (*dark gray*) and receptor site 2′ are represented by the FnIII-1 domain (*light gray*). *B*, crystal structure of IGF-1 (PDB code 5U8Q from Ref. 19, in *violet*) bound to L1 domain (in *light gray*) and αCT (in *dark gray*) representing site 1′ of IGF-1R. *C*, NMR structure of human IGF-2 (PDB code 5L3L from Ref. 26, in *green*). The side chains of residues modified in this study are shown as *sticks* and are *numbered*.

Crystallographic studies provided structural details about interactions of site 1 of insulin (16, 17) or IGF-1 (18, 19) with site 1′ formed by L1 and α-CT domains of IR-A or IGF-1R. Interactions of insulin and IGF-1 with site 1′ of the receptors are similar (Fig. 2, *A* and *B*). The results confirmed the conclusions of previous mutational studies with site 1 of insulin (reviewed in Ref. 20). To date, no structural information is available for a complex of IGF-2 (Fig. 2*C*) with either insulin or IGF-1 receptor, but it can be expected that at least site 1–site 1′ interactions will be similar to insulin and IGF-1.

Characterization of site 2–site 2′ contacts has resisted all attempts in the long term for structural analyses, probably because of the highly dynamic character of the interaction. Recently, two studies revealed the character of site 2–site 2′ binding of insulin with IR-A. Scapin *et al.* (21) published a cryo-EM structure of the insulin receptor extracellular ectodomain in a complex with insulin. The results showed that site 2 in insulin is structurally restricted to Thr-A8, Cys-A7, Gln-B4–Gly-B8, and His-B10 residues, interacting with the receptor's site 2′ FnIII-1 domain. Shortly thereafter, Weis *et al.* (22) confirmed the findings of Scapin *et al.* by solving another cryo-EM structure of insulin bound to the IR-A receptor soluble ectodomain construct. This study extended insulin's site 2 for Glu-B13. However, these structural results do not fully match the results of mutagenesis and kinetic studies with insulin, which indicated that amino acids Ile-A10, Ser-A12, Leu-A13, and Glu-A17 should form insulin's site 2 as well (15, 20).

Therefore, we focused on insulin residues Ile-A10, Ser-A12, Leu-A13, and Glu-A17 and prepared a series of mutants to study their interactions with the receptors. Each hormone residue investigated in this study was mutated with two different amino acids. First, all mutated positions were substituted for His. Histidine has a relatively large side chain, and its imidazole group with a partially aromatic character and ability of hydrogen bonding can participate in different protein–protein interactions. Hence, a rather significant impact on the binding and activation properties of analogs can be expected in the case of His mutations. Second, each of the modified residues was also mutated with a similar amino acid (*e.g.* Ser to Thr, etc.). Here, a subtler modulation of the properties of hormones can be expected. In parallel, similar mutations were prepared in homologous positions of IGF-1 (positions Ser-51, Asp-53, Leu-54, and Glu-58) and IGF-2 (positions Ser-50, Asp-52, Leu-53, and Glu-57) (Fig. 1). The 3D structures of the hormones with highlighted residues modified in this study are shown in Fig. 2.

We determined the binding affinities of prepared mutants for IR-A, IR-B, and IGF-1R and the abilities of mutants to activate these receptors. Computational simulations provided information about the roles of mutations in insulin structure. We compared the receptor-bound structure of native IGF-1 with the NMR structure of a less active Asp-58–IGF-1 mutant. The results allowed direct comparison of equivalent sites in the hormones and provided an unusually complex view of the roles of mutated residues in binding and activation of the receptors.

## Results

### Design and production of analogs

Three series of hormone analogs with single mutations were planned: insulin analogs modified at the positions Ile-A10, Ser-A12, Leu-A13, and Glu-A17; IGF-1 analogs modified at the positions Ser-51, Asp-53, Leu-54, and Glu-58; and IGF-2 analogs modified at the positions Ser-50, Asp-52, Leu-53, and Glu-57. These positions are structurally equivalent in all three hormones (Figs. 1 and 2), and based on the results of several mutagenesis studies with insulin (Ref. 20 and the references therein), IGF-1 (23), and IGF-2 (24, 25), they were considered as parts of hormones' sites 2. We intended to mutate each position in two ways. We either introduced a homologous exchange; *i.e.* Ser → Thr, Leu(Ile) → Val, Glu → Asp, and Asp → Glu or exchanged the WT amino acid for His. The planned analogs are listed in Table 1.

**Table 1:**
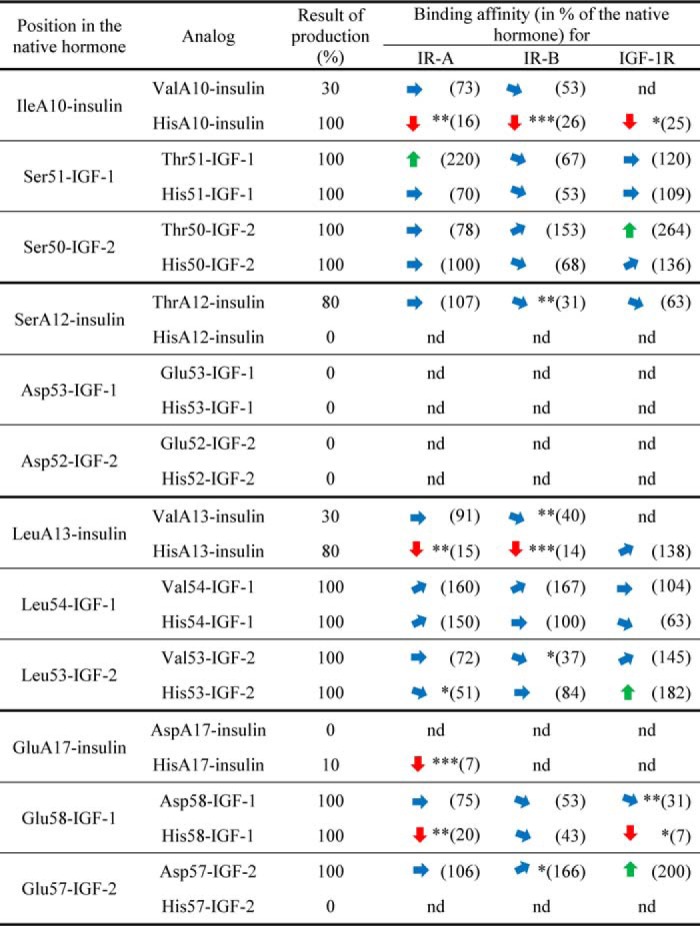
**Simplified overview of relative receptor-binding affinities of insulin, IGF-1, and IGF-2 analogs** The relative binding affinities are shown in % of the native hormone, which has 100% binding affinity for the specific receptor (*i.e.* insulin analogs are related to human insulin, IGF-1 analogs are related to IGF-1, etc.). The approximate major trends in binding affinities of the analogs are indicated by arrows: the upward greeen arrow means >170%, upward diagonal blue arrow means 170–130%, sideways blue arrow means 130–70%, downward diagonal blue arrow 70–30%, and downward red arrow <30% of binding affinity of the native hormone (100%). Numbers in parentheses show mean *K_d_* values, nd is not determined. Asterisks indicate that binding of the ligand to the receptor by the ligand differs significantly from that of the native hormone. *, *p* < 0.05; **, *p* < 0.01; ***, *p* < 0.001. Details are provided in Tables S1–S3. The results of production are related (in %) to native hormones. The typical approximate yield for standard chemical synthesis of insulin (starting with 100 μmol of resin) was ∼ 1 mg. The typical yield for IGF-1 or IGF-2 production from 1 liter of medium was ∼ 0.4 or 0.3 mg, respectively.

Insulin analogs were prepared by the solid-phase peptide synthesis of A and B chains and biomimetic recombination of their disulfide bridges (27–29). IGF-1 and IGF-2 analogs were prepared in *Escherichia coli* cells as previously (26, 30). All IGF-1 analogs have an extra glycine at the N terminus, which allowed cleavage of the fusion protein by TEV protease. We have already shown (30) that the extra Gly residue at −1 position of the hormone does not affect the receptor-binding properties of analogs.

It seems that positions A12/53/52 are important for folding of all three hormones, because only Thr-A12–insulin was prepared. In addition, modifications of the A17 position in insulin and the equivalent 57 position in IGF-2 (but not 58 in IGF-1) also did not produce all the planned analogs, which indicates some roles in insulin/IGF-2 folding. On the other hand, all analogs modified at positions A10/51/50 and A13/54/53 were produced, although yields of Val-A10–insulin and Val-A13–insulin were not sufficient for all biological experiments (Table 1). Table 1 shows production yields of insulin and IGF analogs related to native hormones. The typical yield for standard chemical synthesis of insulin (starting with 100 μmol of resin) was ∼1 mg. The typical yield for IGF-1 or IGF-2 production from 1 liter of medium was ∼0.4 or 0.3 mg, respectively. IGF-1 and IGF-2 analogs were prepared in at least two independent experiments. More laborious chemical synthesis of insulin analogs was performed only once in each case.

### Receptor-binding affinities and receptor activation abilities of analogs

All produced hormone analogs were tested for their binding to IR-A, IR-B, and IGF-1R receptors and for their ability to induce phosphorylation of these receptors. The binding data are summarized in Table 1, the detailed results of biological experiments are provided in Tables S1–S3, representative binding curves of analogs for receptors are shown in Figs. S1–S3, and representative Western blots for relative abilities of analogs to stimulate receptors' phosphorylation are shown in Figs. S4–S6.

Some general trends in binding affinities are clearly visible, despite the fact that not all planned analogs were produced and tested with all the receptors. For positions A10/51/50, Binding of His-A10–insulin is severely compromised for all three receptors. On the contrary, all IGF-1 and IGF-2 analogs modified at these positions are tolerated. For positions A12/53/52, the only successfully prepared Thr-A12–insulin with homologous Ser-to-Thr mutation has binding affinities similar to native insulin for IR-A and IGF-1R but statistically significantly decreased for IR-B receptor. For positions A13/54/53, similarly to A10 position, His-A13 significantly reduces binding affinity of the analog for both isoforms of IR and all IGF-1 and IGF-2 analogs with mutations at equivalent positions have binding properties generally similar to the native hormones. However, on the contrary to His-A10–insulin, His-A13–insulin has a native-like affinity for IGF-1R. For positions A17/58/57, mutations at these positions provided a more complicated picture of the binding affinities of analogs. His-A17–insulin, similarly to A10 and A13 positions, has a very low binding affinity for IR-A. No binding data are available for IR-B and IGF-1R because of the low amount of the analog prepared, but we were able to show that His-A17–insulin is inactive in inducing IGF-1R autophosphorylation (Table S3), which indicates that its IGF-1R binding is severely impaired as well. Interestingly, homologous Glu-to-Asp mutations at position 58 in IGF-1 and position 57 in IGF-2 resulted in different biological effects. Asp-57–IGF-2 binds in a similar manner as native IGF-2 to IR-A/IGF-1R and slightly better to IR-B. On the other hand, Glu-to-Asp change at position 58 of IGF-1 had a rather reducing effect on the analog's binding affinity for IGF-1R. This trend was confirmed by the very low binding affinity of His58-IGF-1 for IGF-1R and by reduced affinities for IR-A and IR-B.

### Activation of receptors

For all prepared analogs, we did not observe any major discrepancies between their binding (*K_d_* values) and trends in receptor activation abilities (Tables S1–S3). We did not detect any important signs of partial or complete antagonism or receptor overactivation.

### NMR structure of Asp58-IGF-1 helped to explain analog's reduced binding affinity

**Figure 3.**
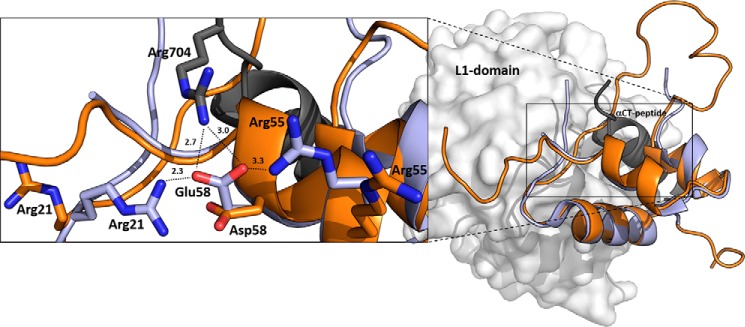
**An overlay of IGF-1R–bound human IGF-1 with Asp-58–IGF-1.** Human IGF-1 is in *light blue* (PDB code 5U8Q from Ref. 19), and a representative (lowest energy) NMR structure of Asp-58–IGF-1 is in *orange* (PDB code 6RVA). The receptor site 1′ is represented by L1 domain (in *gray*) and α-CT peptide (in *black*). The enlarged window on the *left* shows side chains of hormones' Glu-58, Asp-58, or Arg-704 (from α-CT) and two other IGF-1 arginines (Arg-21 and Arg-55) as *sticks* with nitrogen atoms in *blue* and oxygen atoms in *red*. Some possible interactions of Glu-58 and Arg-704, Arg-21, and Arg-55 residues identified in the complex are indicated by *dashed lines* with distances in Å.

We determined the NMR structure of ^15^N- and ^13^C-labeled Asp58-IGF-1 to investigate the interesting effects of Glu-to-Asp substitution at the 58 position of IGF-1. The representative (lowest energy) NMR structure of Asp-58–IGF-1 analog (PDB code 6RVA; this work) was compared with the IGF-1R receptor-bound crystal structure of native IGF-1 (PDB code 5U8Q from Ref 19). Fig. 3 shows that the structures of the Asp-58–IGF-1 analog and receptor-bound native IGF-1 are very similar. The structure similarity of Asp-58–IGF-1 and native IGF-1 is supported by the observed small differences of NMR chemical shifts NH, ^15^N and Hα (supporting information). The only marked differences are in the C domain that is known to be flexible and thus only partly visible in the complex and is probably rearranged during binding to the receptor.

The structure of the IGF-1·IGF-1R complex (PDB code 5U8Q) shows that α-CT's Arg-704 side chain points to Glu-58 carboxylate of complexed IGF-1 and that these residues could possibly form a salt bridge. Some further potential intramolecular stabilization of receptor-complexed native IGF-1 Glu-58 residue could also be deduced from the positions of two other Arg-21 and Arg-55 residues from IGF-1. Locking of Glu-58 in place to enable this salt bridge could be maintained by two intramolecular salt bridges from Arg-21 and Arg-55 residues from IGF-1 (Fig. 3). These latter interactions are expected to be weak and transient, because the electron densities for the two Arg side chains are missing in PDB code 5U8Q. Moreover, the crystallographic resolution of the IGF-1R·IGF-1 structure is 3.27 Å (PDB code 5U8Q) (19), which means that interpretations should be made carefully.

The Asp-58 side chain of the less active Asp-58–IGF-1 analog is one methylene shorter than the Glu-58 side chain. Consequently, Asp-58 may have difficulties to maintain all three salt bridges possibly present in the complex. Indeed, modeling of the complex (not shown) suggests that a rearrangement of the IGF-1 and/or α-CT helix would be needed to enable a potential Asp-58–Arg-704 salt bridge. The stabilization of Asp-58 in IGF-1 by intramolecular contacts with Arg-21 and Arg-55 is improbable (at least in solution) because it is not present in any of 20 available NMR structures (PDB code 6RVA).

### Insulin analogs' metadynamics reveals different free energy profiles that can affect site 1 binding

**Figure 4.**
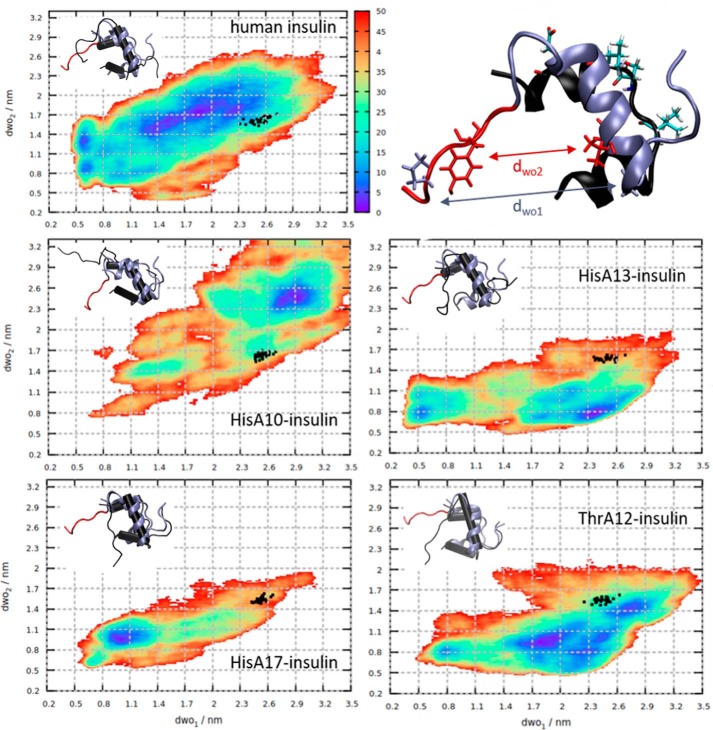
**Free energy profiles (*color scale* in kJ/mol) of human insulin and analogs obtained from metadynamics.** *Black dots* represent insulin conformations from molecular dynamics simulations in complex with IR. The B-chain of representative minimal energy conformers of insulin mutants (in *black*) aligned to the IR-bound conformation of human insulin (*ice blue*, C terminus in *red*) are depicted in the *insets*. The IR-bound conformation of human insulin is shown on the *upper right*, with residues defining the dwo_1_ (Gly-B8–Pro-B28) and dwo_2_ (Val-B12–Tyr-B26) distances, represented as *ice blue* and *red licorice*, respectively. The A-chain is in black, with the mutated residues (Ile-A10, Ser-A12, Leu-A13, Glu-A17) shown as *licorice* and colored by atom type.

Because the insulin residues Ile-10, Ser-A12, Leu-A13, and Glu-A17 were not found to be directly involved in binding the insulin receptor (21, 22), we tested the possibility that mutations at these sites can affect the dynamics of insulin analogs, especially the crucial detachment of the B-chain C terminus of insulin (17, 31), that could in turn modulate binding to the receptor. The native human insulin along with the His-A10, His-A13, and His-A17 analogs with impaired affinity and the native-like affinity Thr-A12 analog (Table 1) were subjected to enhanced-sampling molecular dynamics simulation in explicit solvent along two collective variables, dwo_1_ (Gly-B8–Pro-B28) and dwo_2_ (Val-B12–Tyr-B26), described before (32), defining the B-chain C terminus opening. Fig. 4 shows that the dynamics of the low-affinity His mutants are qualitatively different in that they are less likely to assume the conformation with the detached B-chain C terminus compatible with IR binding.

The WT insulin is likely to assume a wide range of open conformations, with the global minimum exceeding the defined threshold for the wide-open state observed by (32). In comparison, the minimal energy ensembles of the His-A10– and the His-A13–insulin are partially collapsed, the B-chain α-helix unwinds locally, and the coil-like structure results in open states. This loss of secondary structure, however, is not compatible with IR binding. On the other hand, the His-A17-insulin is stabilized in a closed conformation, with both the N and C termini of the B chain remaining close to the insulin core. Here, no detachment of the B-chain C terminus occurs, and thus no IR binding can be expected. Interestingly, the native-like affinity Thr-A12–insulin shows a free energy profile similar to the native insulin where both a broad range of wide-open conformations are possible and the structural integrity of the B-chain α-helix is not as compromised.

Because all mutated sites are distant from the B-chain C terminus segment, we were interested in identifying the specific inter-residue contacts that form in the course of biasing the B-chain C terminus opening and destabilizing the protein core. We might expect the mutations at apolar sites Ile-A10 and Leu-A13 to a polar hydrogen-bond donor/acceptor histidine to be disruptive.

It is apparent from the minimal energy ensemble (in Fig. 4), the His-A10–insulin mutant underwent the most pronounced collapse, resulting in the partial disintegration of the B-chain α-helix. Similarly, the destabilization of the His-13–insulin mutant was mostly restricted to the B chain, although not as pronounced. The difference in contact lifetimes between the mutant and WT insulin revealed that contacts between a range of B-chain N-terminal residues and residues in the α-helix were established to maintain these partially collapsed states (Fig. S7). The low-affinity His-A17–insulin was stabilized in the closed conformation with the N terminus of the B-chain placed parallel to the B-chain α-helix but also close to the A-chain termini (Fig. 4). On the other hand, although the native-like affinity Thr-A12 mutant was stabilized in open conformations, contact analysis revealed that hydrophobic contacts at the A/B chains' interface of insulin more frequently formed than in the WT. This compaction of the hydrophobic core was observed for all other mutants as well, except for the His-10–insulin, which collapsed to more extended states (Fig. S8*A*). Connected to the extent of the hydrophobic collapse, we observed a change in protein–water hydrogen bonding local to the point of mutation. The dynamics of H-bond formation were more stable in the closed compact state of His-A17 mutant (Fig. S8*B*). On the other hand, the bulkier Thr-A12 was buried inside the core and formed fewer H-bonds with water than the WT Ser-A12.

Having considered the conformational diversity of the minimal energy ensembles of insulin mutants, we might expect the conformational strain necessary to assume the insulin–IR–bound form to contribute to the mutation-induced changes in affinity. Consequently, we estimated the free energy of strain upon binding as the relative free energy going from insulin free in solution, taken as the global minimum in Free Energy Surface (dwo_1_, dwo_2_) in Fig. 4, to insulin bound to the IR, corresponding to the *black dots* in Fig. 4 obtained from molecular dynamics of insulin–IR complexes. The insulin mutant–IR–bound complexes were produced starting from the cryo-EM structure of the WT human insulin bound to the IR-A isoform of the receptor (22) and mutagenesis of the respective sites in the A chain (see “Experimental procedures”). The resulting free energies of strain of His-A10, His-A13, His-A17, and Thr-A12 mutants relative to native insulin were 11.5 ± 1.8, 23.7 ± 1.8, 21.2 ± 1.9, and 8.0 ± 3.8 kJ mol^−1^, respectively (the absolute value for the free energy of the strain of native insulin was 16.1 ± 2.8 kJ mol^−1^). The experimentally measured relative binding free energies between insulin mutants and native insulin evaluated using ΔΔ*G*_exp_ = RTln(*K_d_*_mutant_/*K_d_*_insulin_) were 4.6 ± 1.2, 4.7 ± 0.1, 6.6 ± 0.4, and −0.2 ± 1.4 kJ mol^−1^, respectively (relative affinities are reported in Table S1). The largest calculated strains of His-A13 and His-A17 and the low strain of Thr-A12 are in qualitative agreement with the total experimentally determined affinities of insulin mutants. We can thus attribute part of the changed IR-binding affinity for the insulin mutants to the change in conformational strain upon IR binding.

## Discussion

Each hormone residue investigated in this study was mutated with two different amino acids, with His and with a similar amino acid (*e.g.* Ser to Thr, etc.). For His mutations, a rather significant impact on the binding and activation properties of analogs was expected. On the other hand, a subtler modulation of the properties of hormones was expected for similar mutations. Interestingly, both these presumptions were confirmed in this study: His-insulin mutants have important effects on binding affinities, and Glu-to-Asp mutation provided differential results in IGF-1 and IGF-2 (Table 1).

Our results indicate that mutations at the positions A12/53/52 in all three hormones, at position A17 in insulin and at position 57 in IGF-2 (but not at equivalent 58 position in IGF-1) can affect conformation of the premature polypeptides and consequently their folding. Our results are relatively surprising, because others were more successful in modifications of A12 and A17 positions in insulin (33), position 53 in IGF-1 (34), and positions 52 and 57 in IGF-2 (24, 25). The reasons for these different production yields could be in different production strategies, *e.g.* yeast *versus E. coli*, different fusion protein partners, etc.

Other studies (cited in Ref. 20) showed that mutations of insulin positions A10, A12, A13, and A17 can negatively affect binding of analogs to IR, and the residues were proposed to belong to the hypothetical site 2 of insulin. Some analogs were prepared for equivalent positions in IGF-1 and IGF-2 as well. A summary of available literature data on receptor-binding affinities of insulin and IGFs is provided in Table S4, but the list does not provide complete information about binding affinities of mutants with all three receptors, *i.e.* IR-A, IR-B, and IGF-1R. In this respect, our study offers a unique, complex, and comprehensive picture of the involvements of mutated residues in hormone–IR-A/IR-B/IGF-1R interactions.

We prepared only Thr-A12–insulin in the A12/53/52 series. Hence, it is not possible to deduce important conclusions about the roles of these positions in receptor binding. Binding affinities of Thr-A12–insulin were similar to the native hormone for all three receptors (Table 1). Taken together, our data and the data of others (Table S4) rather do not indicate any crucial roles of A12/53/52 positions in binding to the receptors.

On the other hand, the results with other mutants revealed interesting differences in the receptor-binding behavior of the hormones. Whereas positions 51/50 and 54/53 in IGF-1/IGF-2 are relatively tolerant to modifications, insulin binding to all three receptors is severely impaired by modifications at A10. However, Leu-to-His mutation of insulin's A13 negatively affects only binding to IR-A and IR-B and not to IGF-1R. This finding is in full agreement with data published by Schäffer (15) (Table S4). First, these data could indicate that insulin and both IGFs on the other hand do not interact with the receptors by the same mechanisms. Second, the difference in IR and IGF-1R responses to mutation at the insulin A13 position could mean that respective sites 2′ in IR and IGF-1R are different. Such a possibility has already been mentioned by others (35–37) and by our team as well (30).

Insulin binding to IR-A and IGF-1R (according to IGF-1R activation; Table S3) was impaired by mutation at A17. Similar results were found for mutations in IGF-1, but not in IGF-2. Notably, the difference in effects of homologous Glu-to-Asp mutations in IGF-1 and IGF-2 is interesting. The closer look at the crystal structure of IGF-1–IGF-1R complex (PDB code 5U8Q) recently reported by Xu *et al.* (19) reveals that Glu-58 could create close contact (2.7–3.0 Å, probably a salt bridge) with Arg-704 of α-CT peptide (Fig. 3). We are aware that this interaction should be considered with caution because of the resolution of the complex (3.27 Å). Furthermore, a different orientation of Asp-58 side in the NMR structure of Asp-58–IGF-1 compared with the position of Glu-58 in complexed IGF-1 (Fig. 3) may be caused by the low pH of the NMR experiment, which would result in a higher probability of protonation of Asp-58. However, it seems logical that the lower binding affinity of Asp-58-IGF-1 could be explained by the inability of the analog's shorter Asp side chain to form these salt bridges, and the very low affinity of His-58–IGF-1 could be explained by a mutual repulsion of His-58 and Arg-704. Interestingly, similar stabilization of homologous insulin's Glu-A17 is not visible in available insulin–IR-A complexes (PDB code 4OGA (16) or PDB code 6HN5 (22)) or in the complex of IGF-1 with IR L1 and IGF-1R α-CT (PDB code 4XSS) (18).

There are no structural data showing a complex of IGF-2 with any of the receptors, which could explain the enhancing effect of Asp-57 in IGF-2 in binding affinity. Hence, the mechanisms of how Glu at A17/58/57 positions in insulin and both IGFs affect hormone binding to receptors are not fully clear. Nevertheless, our data indicate that at least Glu-58 in IGF-1 can interact with α-CT Arg-704 and that would classify Glu-58 as a part of hormones' site 1.

It cannot be excluded that mutations at insulin A10/A12/A13/A17 sites can affect the structure of the hormone and consequently its ability to interact with receptors' site 1′. To investigate this possibility, we initiated a series of computational experiments with insulin mutants and with native insulin. Native insulin receptor-bound conformation is characterized by a partial detachment of B25–B30 residues from the core of the insulin molecule that is essential for potent insulin binding to receptor site 1′ (17, 38, 39). The data summarized in Fig. 4 show that His mutations at A10/A13/A17 (but not Ser-to-Thr mutation at A12) could have an impact on insulin conformational dynamics and negatively affect the ability of mutants to adopt what are called “active” (open) conformation at insulin's site 1. The theoretical data are in good general agreement with the binding data of analogs. Therefore, as an alternative to the hypothesis that insulin residues A10, A13, and A17 are involved in direct interaction with the receptor site 2, we suggest that they may be important for the structural integrity of the hormone at its site 1.

It is important to bear in mind the advantages and limitations of the computational protocol employed. The benefit of metadynamics (40) as opposed to classical molecular dynamics is its efficiency in accelerating slow conformational transitions along the selected collective variables, while having control over statistical convergence. On the other hand, the added bias may induce unnatural conformational states. Balancing these opposing effects constitutes a demanding project, which is beyond the current study. For the sake of very rough error boundaries, we have, however, carried out several calculations differing in simulation length, biasing criteria, and the initial structure and found uncertainties in the strain-free energies of 10–20 kJ·mol^−1^. This effect is of a greater order of magnitude than the statistical error bounds presented above. With that in mind, the current computational results allow for putting forward an alternative hypothesis of the effect of insulin mutations in the A chain, which will warrant further study.

Weis *et al.* (22) proposed that insulin A12, A13, A17, and B17 residues, predicted by mutagenesis studies to belong to hormone site 2 but not found in contacts with the receptor site 2′ in cryo-EM insulin–IR complexes, may be involved in the initial docking of insulin to the receptor, an event postulated to precede the relaxation of insulin's induced fit to its primary binding site (19). This hypothesis could also explain our experimental data showing that mutations at insulin positions A10, A13, and A17 can cause important changes in receptor-binding affinities that can even be receptor-specific (for the A13 position). Interestingly, insulin analogs mutated at Leu-A13 were shown to have slow association rates, which supports the hypothesis that A13 residue is involved in some first contacts with the receptor (15, 41). The data also indicate that such hypothetical initial docking interactions of insulin and both IGFs with the receptors could be different, because only positions 58/57 and not positions 51/50 and 54/53 in IGF-1/IGF-2 were sensitive to mutations. It is not excluded that future advances in X-ray crystallography or cryo-EM methodology will decipher structures of such hypothetical transient protein hormone complexes and reveal complex mechanisms of receptor activation by the hormones. In this context, during the preparation and revision of this manuscript, two studies (42, 43) were published showing cryo-EM maps of the IR extracellular domains with four insulins bound. Two of these insulins are positioned as shown previously by Scapin *et al.* (21) but the binding site for the other two insulins is located in the FnIII-1 (or FnIII-1′) domain and was not detected previously. This newly identified binding site was proposed as a new site 2 and should interact with insulin residues studied in this work. These findings support the hypothesis of initial docking and transient interactions between insulin and IR that are followed by structural rearrangements of the complex. These new findings are not contradictory to our results because mutations of insulin residues A12, A13, and A17 could affect both insulin dynamics and site 1 interactions, as well as new site 2 interactions.

## Conclusions

Deciphering molecular mechanisms by which hormones insulin, IGF-1, and IGF-2 bind their cognate receptors and elicit different biological effects has been a central problem for biologists for decades. Two cryo-EM studies (21, 22) showed how insulin binds the insulin receptor through its binding sites 1 and 2. However, these findings do not fully match the results of mutagenesis studies, which predicted that insulin residues Ile-A10, Ser-A12, Leu-A13, and Glu-A17 should belong to hormone site 2 as well. Therefore, we systematically mutated these hypothetical insulin site 2 residues and equivalent residues in IGF-1 and IGF-2. Comparison of the biological properties of insulin, IGF-1, and IGF-2 site 2 mutants on three transmembrane receptors (IR-A, IR-B, and IGF-1R) revealed that the hormones respond to equivalent mutations differently and that responses can be receptor-specific. Specifically, we showed that insulin sites A10 and A17 are important for binding to all tested receptors, but A13 is only important for IR-A and IR-B. In IGF-1/IGF-2, the positions 51/50 and 54/53 probably do not play any important role in receptor binding. On the other hand, we propose that Glu-58 of IGF-1 can interact with the IGF-1R site 1 Arg-704 residue, and hence Glu-58 could belong to IGF-1's site 1. The results of computational metadynamics show that mutations can affect the internal dynamics of insulin and inhibit its ability to adopt receptor-bound conformation, which is important for binding to receptor site 1. This indicates that studied insulin residues might not be involved in direct interactions with site 2 of receptors. Recently, two studies (42, 43) were published showing cryo-EM maps of the IR extracellular domains with insulin bound to a newly identified binding site that was proposed as a new site 2 and should interact with the insulin residues studied in this work. These new findings support the hypothesis of initial docking and transient interactions between insulin and IR.

## Experimental procedures

### Synthesis of insulin analogs

Insulin analogs were prepared by the solid-phase chemical synthesis of A and B chains in their S-sulfonate forms, followed by a biomimetic recombination of disulfide bridges according to previously published protocol (27, 29). The peptide synthesis was performed on a Spyder Mark II automatic peptide synthesizer (a prototype developed by a team in the developmental workshops in the Institute of Organic Chemistry and Biochemistry, headed by Dr. Michal Lebl; the European patent application number is EP 17206537.7).

### Cloning and production of IGF-1 and IGF-2 analogs

IGF-1 and IGF-2 analogs were produced according to our previously published methodology (26, 30). Briefly, both human IGF-1 (UniprotKB entry P05019 amino acids 49–118) and human IGF-2 (UniprotKB entry P01344 amino acids 25–91) were cloned into a modified pRSFDuet-1 expression vector as a fusion with an N-terminally His_6_-tagged GB1 protein and TEV protease cleavage site. An additional N-terminal glycine residue (Gly^−1^) was incorporated into IGF-1 to enable cleavage by TEV protease (sequence Glu-Asn-Leu-Tyr-Phe-Gln↓Gly^−1^), but the Glu-Asn-Leu-Tyr-Phe-Gln↓Ala^1^ sequence yielding a native hormone was used for TEV protease cleavage site (↓) in the IGF-2 expression constructs. Constructs were transformed into *E. coli* BL21(λDE3) and cultivated by using LB medium or minimal medium containing [^15^N]ammonium sulfate and [^13^C]d-glucose, and hormones were purified as described previously (26).

All successfully produced analogs were purified by reverse-phase HPLC. The purity of all tested analogs was higher than 95% (and controlled by reverse-phase HPLC analyses and high resolution MS spectra).

### Binding affinities for the receptors

Binding affinities of analogs were determined with receptors in the intact cells. Specifically, binding affinities for IGF-1R were determined with mouse fibroblasts transfected with human IGF-1R and with deleted mouse IGF-1R, according to Hexnerová *et al.* (26). Binding affinities for IR-A were determined with human IR-A in human IM-9 lymphocytes, according to Viková *et al.* (44). Binding affinities for IR-B were determined with mouse fibroblasts transfected with human IR-B and with deleted mouse IGF-1R, according to Záková *et al.* (31). The binding curve of each analog was determined in duplicate, and the final dissociation constant (*K_d_*) was calculated from at least three (*n* ≥ 3) binding curves (each curve giving a single *K_d_* values), determined independently and compared with binding curves for insulin, IGF-1, or IGF-2, depending on the type of analog.

### The abilities of analogs to induce autophosphorylation of the receptors

The abilities of analogs to induce autophosphorylation of IGF-1R in membranes of mouse fibroblast transfected with human IGF-1R and with deleted mouse IGF-1R were determined, as described by Macháčková *et al.* (29). The abilities of analogs to induce autophosphorylation of IR-A or IR-B in mouse fibroblast transfected with human IR-A or IR-B and with deleted mouse IGF-1R were determined, as described by Křížková *et al.* (45). Briefly, the cells were stimulated in 24-well plates (Schoeller) (4 × 10^4^ cells per well) after 4 h of starving in serum-free medium. The cells were stimulated with 10 nm concentration of the ligands (insulin, IGF-1, IGF-2, or analogs) for 10 min. Stimulation was stopped by snap-freezing. Proteins were routinely analyzed, using immunoblotting and horseradish peroxidase-labeled secondary antibodies (Sigma–Aldrich). The membranes were probed with anti-phospho-IGF-1Rβ (Tyr-1135/1136)/IRβ (Tyr-1150/1151) (Cell Signaling Technology). The blots were developed using the SuperSignal West Femto maximum sensitivity substrate (Pierce) and analyzed using the ChemiDoc MP imaging system (Bio-Rad). The autophosphorylation signal density generated by each ligand on Western blotting was expressed as the contribution of phosphorylation relatively to the IGF-1 (IGF-1R fibroblasts) respective human insulin (IR-A and IR-B fibroblasts) signal in the same experiment. Means ± S.D. values were calculated from four independent experiments (*n* = 4) and compared with native insulin, IGF-1, or IGF-2, depending on the type of analog.

The significance of the changes in binding affinities was calculated using the two-tailed *t* test. The significance of changes in abilities of analogs to stimulate autophosphorylation was calculated using one-way analysis of variance with Dunnett's test, comparing all analogs *versus* control (insulin or IGFs, depending on the type of the analogs).

### NMR spectroscopy

All NMR data for Asp-58–IGF-1 and native IGF-1 were acquired on 600 MHz Bruker Avance II spectrometer equipped with a 5-mm ^1^H/^13^C/^15^N cryoprobe. The NMR spectra were collected at 40 °C using 450-μl samples of protein dissolved in 50 mm solution of CD_3_COOD (pH 3.0) in water (95% H_2_O + 5% D_2_O) with 0.01% NaN_3_.

Proton NMR data of both proteins were obtained from homonuclear 2D total correlation spectroscopy and 2D-NOESY spectra of nonlabeled samples: native IGF-1 (0.44 mg; 0.11 mm) and Asp-58–IGF-1 (0.70 mg; 0.18 mm). Isotopically ^15^N-labeled native IGF-1 (0.25 mg; 0.06 mm) and Asp-58–IGF-1 (0.07 mg; 0.02 mm) were used for determination of ^15^N chemical shifts from 2D-^1^H,^15^N-HSQC (heteronuclear single quantum coherence) experiments. Doubly ^13^C,^15^N-labeled Asp-58–IGF-1 (0.18 mg; 0.05 mm) dissolved in D_2_O provided also ^13^C chemical shifts from 2D-^1^H,^13^C-HSQC and 2D-^1^H,^13^C-HMBC (heteronuclear multiple bond coherence) experiments. Structurally assigned ^1^H, ^15^N, and ^13^C NMR data (Tables S5–S7) were then used for 3D structure calculations.

### Structure elucidation

Structures were calculated, using XPLOR-NIH (46) with implicit solvent and default force field. Distance constraints (with tolerances +20%, −50%) were derived from manually picked NOESY cross-peaks using CcpNmr analysis (47). TALOS-N (48) was used to generate backbone dihedral angle restraints from ^1^H, ^13^C, and ^15^N chemical shifts, whereas only predictions classified as “strong” were used. Also, a few *J*(NH,Hα)-based restraints were applied for residues with coupling value of >8 Hz. There were no explicitly enforced hydrogen bonds. After the first rounds of structure calculation, several Cα–Cβ dihedral angle restraints were added that were based on a combination of preliminary structure, NOE contacts, and *J*-coupling values.

Starting with 100 randomly generated extended structures, the simulation protocol consisted of two rounds of simulated annealing. The first round of simulated annealing began with short molecular dynamics at temperature 3500 °C (variable integration time step; 1000 steps or 100 ps, whichever was met first) followed by slow cooling to 25 °C with the 1 °C step (variable integration time step; 100 steps or 0.2 ps at every temperature, whichever was met first.

The structure with the lowest constraint violation count was subsequently selected as the starting structure for the next round of simulated annealing. Distance restraint was considered as violated when the difference between the calculated and the experimental distance was more than 0.3 Å. The starting structure was simulated from temperature 3000 °C to 25 °C with 0.5 °C step (otherwise the same annealing protocol as the previous round). This was repeated with random starting velocities, yielding another 100 different protein conformations, from which 20 structures with no constraint violations were selected and sorted with respect to the force field energy combined with the energy of NOE term. The atomic coordinates of Asp58–IGF-1 analog (PDB code 6RVA) have been deposited in the Protein Data Bank.

### Structural modeling of the IR-insulin complex and equilibration of the system

Starting from the cryo-EM structure (22), we remodeled the missing loops in the original structure, using PyMOL (PyMOL Molecular Graphics System, version 2.0 Schrödinger, LLC). The remodeled loops contained residues 160–168 of the CR domain, 447–455 of the L2 domain, 824–843 of the FnIII-1′ domain and the B-chain residues in insulin, N-terminal B1–B6, and C-terminal B27–B30. Molecular dynamics simulations were carried out using Gromacs 5.1.2 (49) and the AMBER ff14SB force field (50). We continued with solvating the system with 32072 OPC3 waters (51) and adding Na^+^ and Cl^−^ ions to the 0.15 m concentration, with extra 10 sodium ions added to neutralize the system. The system was minimized in 2000 steps with steepest descent. We then heated only the remodeled part of the structure with the rest of the protein kept frozen, with simulated annealing with time increments of 10 ps and temperature increments of 50 K. The sampling of the remodeled loops was enhanced in this way by heating up to 600 K and cooling back to 300 K. The whole system was then equilibrated in an NpT ensemble at 300 K for 500 ps and the pressure of 1 bar. The cutoff distance for the nonbonded interactions was 1.4 nm, with electrostatics treated with particle mesh Ewald and the van der Waals interactions with a simple cutoff. The ending structure of the WT insulin–IR complex was used as a starting structure for a 20-ns production run. For the production of insulin mutant–IR complexes, we used the mutagenesis plugin in PyMOL to introduce His residues (HIE, histidine with hydrogen on the epsilon nitrogen) at A-chain WT sites Ile-10, Leu-13, and Glu-17 and the Thr residue at site Ser-12. These complexes were then minimized and equilibrated for 500 ps, first in the NVT ensemble at 300 K and then in the NpT ensemble to reach the pressure of 1 bar.

### Metadynamics of insulin mutants to enhance sampling of the B-chain C terminus detachment

The structures of insulin monomers were extracted from the minimized and equilibrated structures of insulin mutant-IR complexes described in the previous sections. The monomers were then neutralized by adding sodium atoms (+1 for His-A17 mutant and +2 for all other neutral mutants and WT), solvating with OPC3 waters and adding Na^+^ and Cl^−^ ions up to 0.15 m concentration. We minimized the system with steepest descent and equilibrated for 500 ps in an NpT ensemble by gradually increasing the temperature in 50-K temperature and 10-ps time increments. The cutoff for nonbonded interactions was 1.4 nm, with particle mesh Ewald and a simple cutoff treatment of the electrostatics and van der Waals interaction, respectively. The metadynamics production runs were performed using the PLUMED plugin (52) to Gromacs 5.1.2 (49). As collective variables to describe the B-chain C terminus detachment, we chose the residues Gly-B8–Pro-B28 and Val-B12–Tyr-B26, which we denote as dwo_1_ and dwo_2_, respectively. These two distances were characterized as most indicative of switching from the closed to wide-open state in a previous molecular dynamics study of WT insulin (32). In metadynamics, a history-dependent potential *V*_G_(*s*,*t*) of Gaussian functions of the form

(Eq. 1) $$V_{\text{G}}\left(s,t\right)=\int_{0}^{t}\text{dt}\prime \text{W}\exp\left(-\sum_{\text{i}=1}^{\text{d}}\frac{{\left(s_{\text{i}}\left(R\right)-s_{\text{i}}\left(R\left(t\prime \right)\right)\right)}^{2}}{2\sigma_{\text{i}}^{2}}\right)$$

is added to selected collected variables *s*(*R*), where *W* is the energy rate, and σ_i_ is the width of the Gaussian potential for the *i*th collective variable. We used the adaptive approach of determining the width based on the space for the collective variable covered in time (ADDAPTIVE = DIFF keyword for PLUMED, from Ref. 40). The Gaussian potentials of 2.5 kJ mol^−1^ height were added every 150 steps. The free energy is reconstructed by summing the added Gaussian potentials, assuming

$$\underset{\text{t}\to \infty }{\lim}\;V_{\text{G}}\left(s,t\right)∼-F\left(s\right)$$

The metadynamics simulation was run for a total of 80 ns.

### Estimation of free energy of strain upon binding for insulin from metadynamics free energy plots

The free energy of strain upon binding Δ*F*_strain_ is defined as the difference in free energies of insulin in the insulin–IR–bound state and free insulin in solution, Δ*F*_strain_ = *F*_ins,bound_ − *F*_ins,free_. The strain for insulin in the IR-bound state *F*_ins,bound_ was estimated based on the projection of the (d_wo1_, d_wo2_) distances assumed in the 20-ns molecular dynamics run of the complex on to the free energy map *F* (d_wo1_, d_wo2_) obtained from metadynamics (Fig. 4). The strain for insulin free in solution was taken as the *F* (d_wo1_, d_wo2_) global minimum, resulting in *F*_ins,free_ = 0.

### Differential contact map calculation

The frequency of inter-residue contact formation, defined for pairs of residues as the fraction of time spent in contact during the metadynamics run, was calculated using the CONAN plugin (53). We defined a contact between residues if their centers of mass were within 0.6 nm distance. To evaluate which contacts are formed more or less frequently in mutants compared with the WT, we calculated the difference between total interaction times Δ*f*_ij_ = *f*_ij,wt_ − *f*_ij,mut_, for the (*i*, *j*) residue pair. These are reported as differential contact maps in Fig. S7.

### Hydrogen bond analysis

The number of water–protein hydrogen bonds local to the point of mutation was determined for a shell of the radius 1 nm around the Cα (CA) atom of a mutated residue. With OH and NH groups regarded as donors and oxygen and nitrogen atoms as acceptors, the donor–acceptor cutoff distance was set to 0.35 nm, and the angle of the hydrogen donor–acceptor was set to 30°.

## Author contributions

K. Macháčková, K. Mlčochová, P. P., O. S., M. B., M. F., I. S., L. Ž., and J. J. investigation; K. Macháčková, K. Mlčochová, P. P., K. H., O. S., M. B., A. M., M. L., M. Č., J. R., M. F., K. Mitrová, M. C., J. L., Y. Y., I. S., and L. Ž. methodology; O. S., M. B., A. M., M. L., I. S., L. Ž., and J. J. formal analysis; M. B., A. M., M. L., L. Ž., and J. J. data curation; M. B., M. L., P. H., I. S., L. Ž., and J. J. supervision; A. M., M. L., I. S., and J. J. writing-original draft; A. M., L. Ž., and J. J. writing-review and editing; M. L., P. H., L. Ž., and J. J. validation; P. H. and J. J. funding acquisition; L. Ž. conceptualization; J. J. resources.

## Supplementary Material

Supporting Information

## Footnotes

4 The abbreviations used are:

IGF: insulin-like growth factor
IR: insulin receptor
IGF-1R: IGF-1 receptor
PDB: Protein Data Bank.
