# Supplementary material for: Mutations at hypothetical binding site 2 in insulin and insulin-like growth factors 1 and 2 result in receptor- and hormone-specific responses
Source: J Biol Chem. 2019 Sep 26;294(46):17371–82. doi: 10.1074/jbc.RA119.010072 (PMC6873181; doi:10.1074/jbc.RA119.010072)
Supplement: Supporting Information [file supp_294_46_17371__index.html]

Mutations at hypothetical binding site 2 in insulin and insulin-like growth factors 1 and 2 result in receptor- and hormone-specific responses — Insulin and IGFs respond to equivalent mutations differently — Mutations at hypothetical binding site 2 in insulin and insulin-like growth factors 1 and 2 result in receptor- and hormone-specific responses — Insulin and IGFs respond to equivalent mutations differently — Supporting Information 

# Mutations at hypothetical binding site 2 in insulin and insulin-like growth factors 1 and 2 result in receptor- and hormone-specific responses

## Supporting Information

- Supporting Information (to be published online) - Supporting Information Tables and Figures
