# Supplementary material for: Mutations at hypothetical binding site 2 in insulin and insulin-like growth factors 1 and 2 result in receptor- and hormone-specific responses
Source: J Biol Chem. 2019 Sep 26;294(46):17371–82. doi: 10.1074/jbc.RA119.010072 (PMC6873181; doi:10.1074/jbc.RA119.010072)
Supplement: Supporting Information [file supp_RA119.010072_154125_2_supp_399468_py4bwg.pdf]

SUPPORTING INFORMATION

Mutations at hypothetical binding Site 2 in insulin and insulin-like growth factors 1 and 2 elicit receptor- and hormone-specific responses

Macháčková *et al*.

Table of contents

Table S1	Binding affinities and receptor activation abilities of analogs for IR-A.
Table S2	Binding affinities and receptor activation abilities of analogs for IR-B.
Table S3	Binding affinities and receptor activation abilities of analogs for IGF-1R.
Figure S1	Representative binding curves of analogs for IR-A
Figure S2	Representative binding curves of analogs for IR-B
Figure S3	Representative binding curves of analogs for IGF-1R
Figure S4	Representative Western blots for relative abilities of IGF-1 analogs to activate receptors'
Figure S5	Representative Western blots for relative abilities of IGF-2 analogs to activate receptors'
Figure S6	Representative Western blots for relative abilities of insulin analogs to activate receptors'
Table S4	Summary of available literature data on insulin/IGF-1/IGF-2 analogs modified at A10/51/50, A12/53/52, A13/54/53 and A17/58/57 positions.
Table S5	Chemical shifts of amino acid residues in Asp58-IGF-1 and native IGF-1.
Table S6	Chemical shift differences between Asp51-IGF-1 and native IGF-1.
Table S7	NOEs statistics.
Figure S7	Differential contact maps calculated from metadynamics of the B-chain C-terminus opening for insulin analogs HisA10, ThrA12, HisA13, HisA17.
Figure S8	Structural characterization of the metadynamics ensembles – hydrophobic collapse and hydrogen bond analysis.

Supplementary references

Table S1. Receptor-binding affinities of human insulin, IGF-1, IGF-2 and analogs for human IR-A in membranes of IM-9 lymphocytes and relative abilities of the hormones/analogs to stimulate phosphorylation of human IR-A in membranes of transfected mouse fibroblasts (details are provided in Methods). The K_d values or relative stimulations were obtained from at least three measurements. (n) is number of replicates. Asterisks indicate that binding of the ligand to the receptor or activation of the receptor by the ligand differs significantly from that of the native hormone (*p<0.05; **p<0.01; ***p< 0.001).

Native hormone or analog	K_d ± S.D. for human IR-A [nM] (n)	Relative binding affinity [a] for human IR-A [%]		Relative stimulation of human IR-A [%]	
Insulin	0.12 ± 0.04 (3)¶	100 ± 33 [b]		100 ± 8	
	0.32 ± 0.10 (4)"	100 ± 31			
	0.30 ± 0.13 (5)€	100 ± 43			
	0.27 ± 0.01 (5)#	100 ± 4			
	0.18 ± 0.01 (4)$	100 ± 6			
	0.25 ± 0.05 (5)^	100 ± 20			
IGF-1	24.0 ± 11.5 (3)^	1.0 ± 0.5 [c]	100 ± 48 [d]	27 ± 10	100 ± 37 [d]
IGF-2	2.9 ± 0.2 (3)^	8.6 ± 1.8	100 ± 7	63 ± 15	100 ± 24
ValA10-insulin	0.44 ± 0.06 (3)"	73 ± 25		74 ± 9*	
HisA10-insulin	0.74 ± 0.24 (4)¶	16 ± 7.5 **		41 ± 11***	
Thr51-IGF-1	8.1 ± 4.8 (4)$	2.2 ± 1.3	220 ± 170	33 ± 10	122 ± 58
His51-IGF-1	38.7 ± 2.75 (3)#	0.7 ± 0.06	70 ± 35	36 ± 12	133 ± 66
Thr50-IGF-2	2.7 ± 0.2 (3)$	6.7 ± 0.6	78 ± 18	68 ± 19	108 ± 40
His50-IGF-2	2.1 ± 0.2 (3)$	8.6 ± 0.9	100 ± 23	70 ± 17	111 ± 38
ThrA12-insulin	0.28 ± 0.11 (3)€	107 ± 62		93 ± 17	
ValA13-insulin	0.35 ± 0.07 (4)"	91 ± 34		71 ± 7*	
HisA13-insulin	2.0 ± 1.0 (3)€	15 ± 9**		33 ± 9***	
Val54-IGF-1	11.2 ± 5.4 (3)$	1.6 ± 0.8	160 ± 113	27 ± 11	100 ± 55
His54-IGF-1	12.2 ± 1.5 (3)$	1.5 ± 0.2	150 ± 78	19 ± 4	70 ± 30
Val53-IGF-2	2.9 ± 0.2 (3)$	6.2 ± 0.5	72 ± 16	40 ± 16	63 ± 29*
His53-IGF-2	4.1 ± 1.5 (3)$	4.4 ± 1.6	51 ± 21*	33 ± 11	52 ± 21**
HisA17-insulin	3.8 ± 0.3 (3)#	7.1±0.6***		0.2 ± 0.2***	
Asp58-IGF-1	24.1 ± 2.8 (3)$	0.75 ± 0.1	75 ± 39	30 ± 16	111 ± 72
His58-IGF-1	91 ± 40 (3)$	0.2 ± 0.09	20 ± 13**	4.8 ± 2.3	18±11***
Asp57-IGF-2	2.96 ± 0.28 (3)#	9.1 ± 0.9	106 ± 24	72 ± 17	115 ± 39

[a] Relative binding affinity is defined as (K_d of the native hormone / K_d of analog) x 100 (%).

[b] The K_d of human insulin for IR-A was determined in six independent series of measurements (indexed with ¶, ", €, #, $ and ^).

[c] The individual K_d values of ligands in this column are relative to a corresponding native insulin K_d value (e.g. $ to $, etc.)

[d] In these columns for each IGF analogue their values relative to insulin are then expressed relative to native IGF-1 (100%) for IGF-1 analogs or relative to native IGF-2 (100%) for IGF2 analogs.

Table S2. Receptor-binding affinities of human insulin, IGF-1, IGF-2 and analogs for human IR-B and relative abilities of the hormones/analogs to stimulate phosphorylation of this receptor in membranes of transfected mouse fibroblasts (details are provided in Methods). The K_d values or relative stimulations were obtained from at least three measurements. (n) is number of replicates. Asterisks indicate that binding of the ligand to the receptor or activation of the receptor by the ligand differs significantly from that of the native hormone (*p<0.05; **p<0.01; *** p<0.001).

Native hormone or analog	K_d ± S.D. for human IR-B [nM] (n)	Relative binding affinity [a] for human IR-B [%]		Relative stimulation of human IR-B [%]	
Insulin	0.31 ± 0.08 (4)€	100 ± 26 [b]		100 ± 4	
	0.35 ± 0.06 (3)"	100 ± 17			
	0.39 ± 0.23 (6)$	100 ± 59			
	0.38 ± 0.10 (4)#	100 ± 26			
	0.68 ± 0.28 (5)^	100 ± 41			
IGF-1	224 ± 33 (4)^	0.3 ± 0.13 [c]	100 ± 43 [d]	11 ± 5.2	100 ± 47 [d]
IGF-2	35.4 ± 11.2 (4)	1.9 ± 1	100 ± 52	20 ± 7.4	100 ± 37
ValA10-insulin	0.66 ± 0.37 (4)"	53 ± 31		93 ± 15	
HisA10-insulin	1.2 ± 0.3 (3)€	26 ± 9***		46 ± 13***	
Thr51-IGF-1	173 ± 18 (3)$	0.2 ± 0.12	67 ± 49	nd	
His51-IGF-1	245 ± 130 (3)$	0.16 ± 0.13	53 ± 49	10 ± 6	91 ± 69
Thr50-IGF-2	13.3 ± 4.3 (3)$	2.9 ± 2	153 ± 132	19 ± 4	96 ± 49
His50-IGF-2	28.9 ± 14.7 (3)$	1.3 ± 1	68 ± 63	25 ± 5	123 ± 52
ThrA12-insulin	1.22 ± 0.30 (3)#	31 ± 11**		63 ± 17*	
ValA13-insulin	0.94 ± 0.15 (3)#	40 ± 12**		60 ± 6**	
HisA13-insulin	2.64 ± 0.61 (3)#	14 ± 4.9***		29 ± 10***	
Val54-IGF-1	78 ± 37 (3)$	0.5 ± 0.38	167 ± 146	13 ± 7	116 ± 83
His54-IGF-1	143 ± 93 (4)$	0.3 ± 0.26	100 ± 97	12 ± 4	111 ± 64
Val53-IGF-2	58 ± 25 (3)$	0.7 ± 0.5	37 ± 33*	14 ± 2	69 ± 27
His53-IGF-2	24.9 ± 9.9 (3)$	1.6 ± 1.1	84 ± 73	6.7 ± 1	33 ± 13**
HisA17-insulin	nd			nd	
Asp58-IGF-1	250 ± 223 (2)$	0.16 ± 0.17	53 ± 61	8 ± 4	73 ± 50
His58-IGF-1	311 ± 74 (3)$	0.13 ± 0.08	43 ± 32	7.8 ± 2.9	71 ± 43
Asp57-IGF-2	9.8 ± 2.8 (6)€	3.16 ± 1.2	166 ± 107*	27 ± 11	135 ± 74

[a] Relative binding affinity is defined as (K_d of the native hormone / K_d of analog) x 100 (%).

[b] The K_d of human insulin for IR-B was determined in five independent series of measurements (indexed with €, ", $, # and ^).

[c] The individual K_d values of ligands in this column are relative to a corresponding native insulin K_d value (e.g. € to €, etc.).

[d] In these columns for each IGF analogue their values relative to insulin are then expressed relative to native IGF-1 (100%) for IGF-1 analogs or relative to native IGF-2 (100%) for IGF2 analogs.

Table S3. Receptor-binding affinities of human insulin, IGF-1, IGF-2 and analogs for human IGF-1R and relative abilities of the hormones/analogs to stimulate phosphorylation of this receptor in membranes of transfected mouse fibroblasts (details are provided in Methods). The K_d values or relative stimulations were obtained from at least three measurements. (n) is number of replicates. Asterisks indicate that binding of the ligand to the receptor or activation of the receptor by the ligand differs significantly from that of the native hormone (*p<0.05; **p<0.01; *** p<0.001).

Native hormone or analog	K_d ± S.D. for human IGF-1R [nM] (n)	Relative binding affinity [a] for human IGF-1R [%]		Relative stimulation of human IGF-1R [%]	
IGF-1	0.11 ± 0.05 (5)#	100 ± 45 [b]		100 ± 21	
	0.24 ± 0.11 (5)$	100 ± 46			
	0.24 ± 0.10 (5)^	100 ± 42			
	0.25 ± 0.09 (4)€	100 ± 36			
	0.19 ± 0.08 (5)"	100 ± 42			
insulin	292 ± 54 (3)^	0.08 ± 0.036 [c]	100 ± 45 [d]	2 ± 0.1***	100 ± 5 [d]
IGF-2	2.3 ± 1.2 (3)€	11 ± 7	100 ± 63	61 ± 17**	100 ± 28
ValA10-insulin	nd			1.6 ± 0.2	80 ± 11
HisA10-insulin	451 ± 53 (3)#	0.02 ± 0.009	25 ± 16*	1.0 ± 0.3	50 ± 15
Thr51-IGF-1	0.20 ± 0.05 (4)$	120 ± 63		83 ± 15	
His51-IGF-1	0.22 ± 0.01 (4)€	109 ± 50		98 ± 30	
Thr50-IGF-2	0.84 ± 0.17 (4)$	29 ± 14	264 ± 210	60 ± 17	98 ± 39
His50-IGF-2	1.6 ± 0.8 (4)$	15 ± 10	136 ± 125	64 ± 21	105 ± 45
ThrA12-insulin	372 ± 174 (4)"	0.05 ± 0.03	63 ± 47	1.6 ± 0.4	80 ± 20
ValA13-insulin	nd			2.0 ± 0.4	100 ± 21
HisA13-insulin	167 ± 44 (4)"	0.11 ± 0.05	138 ± 88	1.4 ± 0.6	70 ± 30
Val54-IGF-1	0.23 ± 0.04 (3)$	104 ± 51		66 ± 16*	
His54-IGF-1	0.38 ± 0.08 (3)$	63 ± 32		73 ± 14	
Val53-IGF-2	1.5 ± 0.3 (3)$	16 ± 8	145 ± 117	45 ± 15	74 ± 32
His53-IGF-2	1.2 ± 0.4 (4)$	20 ± 11	182 ± 152	46 ± 20	75 ± 39
HisA17-insulin	nd			0	0
Asp58-IGF-1	0.80 ± 0.26 (3)€	31.3 ± 15**		99 ± 32	
His58-IGF-1	3.3 ± 0.2 (3)$	7.3 ± 3.4*		60 ± 15**	
Asp57-IGF-2	0.5 ± 0.2 (4)#	22 ± 13	200 ± 173	73 ± 23	120 ± 50

[a] Relative binding affinity is defined as (K_d of the native hormone / K_d of analog) x 100 (%).
[b] The K_d of human IGF-1 for IR-IGF-1R was determined in five independent series of measurements (indexed as #, $, ^, € and ").
[c] The individual K_d values of ligands in this column are relative to a corresponding native IGF-1 K_d value (e.g. # to #, etc.).
[d] In these columns for each IGF analogue their values relative to insulin are then expressed relative to native IGF-1 (100%) for IGF-1 analogs or relative to native IGF-2 (100%) for IGF2 analogs.

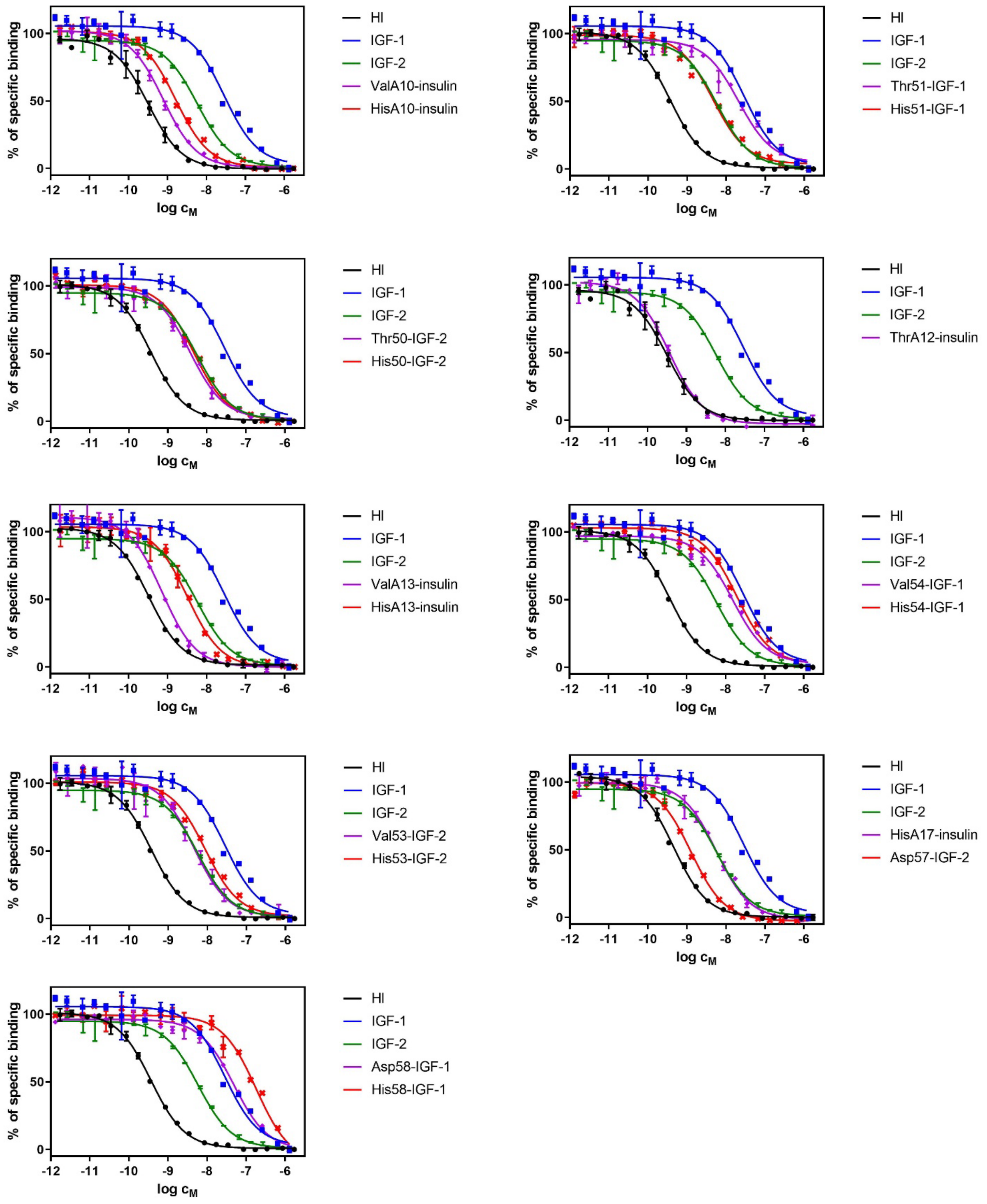

Figure S1. Binding curves for IR-A. Inhibition of binding of human [^{125}I]-monoiodotyrosyl-insulin to IR-A by human insulin (HI), IGF-1, IGF-2 and analogs. Representative binding curve for each hormone or analog is shown.

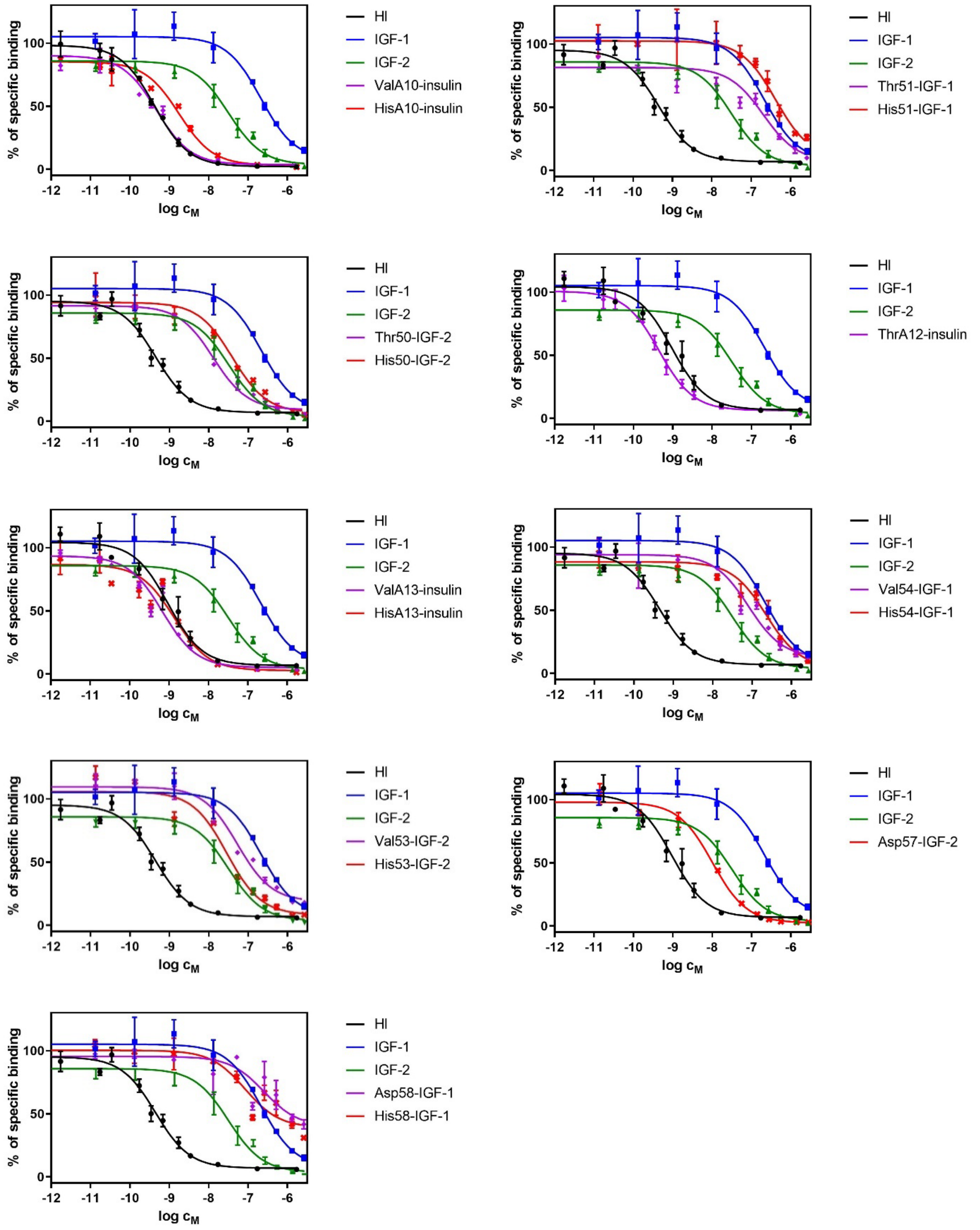

Figure S2. Binding curves for IR-B. Inhibition of binding of human [^{125}I]-monoiodotyrosyl-insulin to IR-B by human insulin (HI), IGF-1, IGF-2 and analogs. Representative binding curve for each hormone or analog is shown.

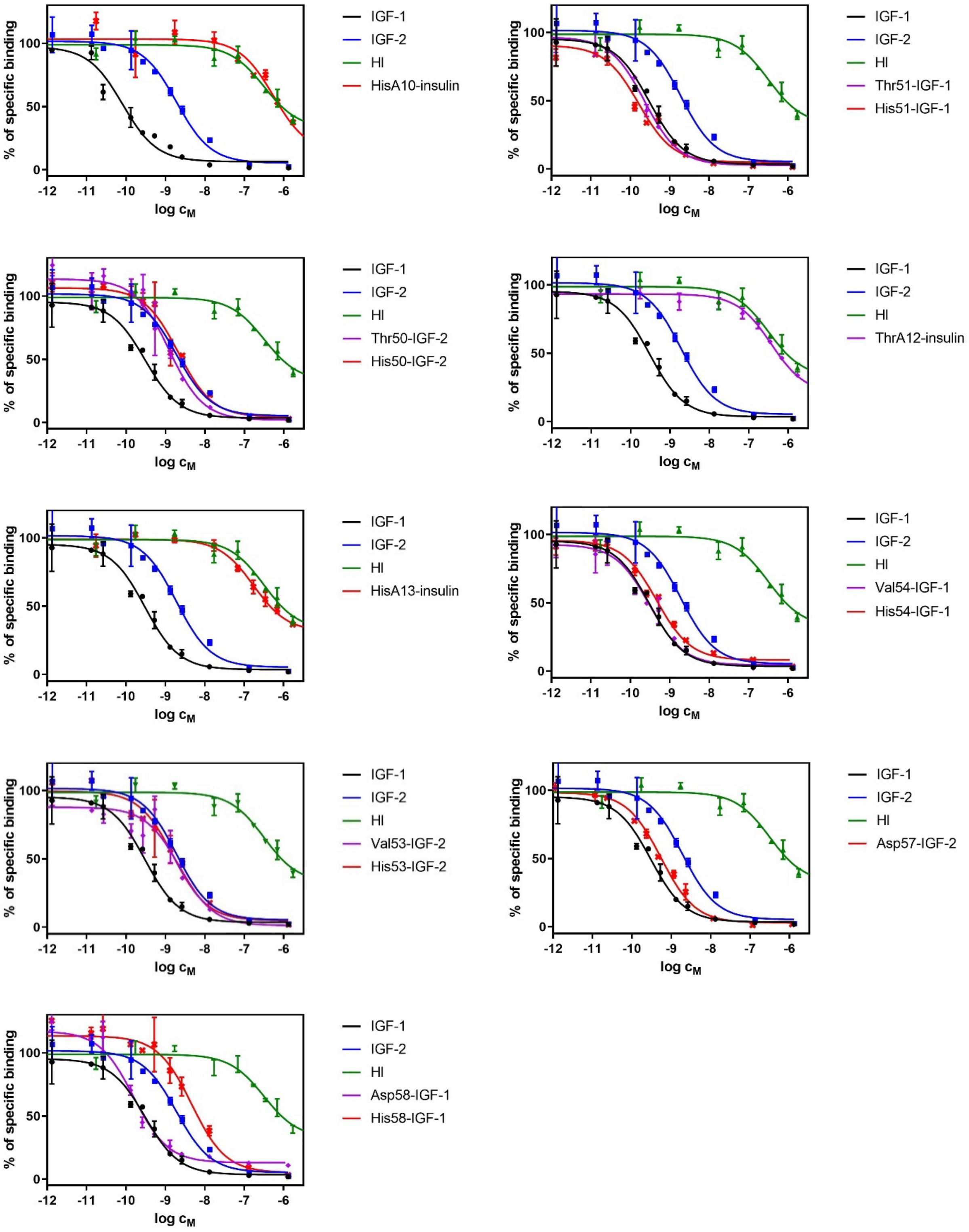

Figure S3. Binding curves for IGF-1R. Inhibition of binding of human [^{125}I]-iodotyrosyl-IGF-1 to IGF-1R by human insulin (HI), IGF-1, IGF-2 and analogs. Representative binding curve for each hormone or analog is shown.

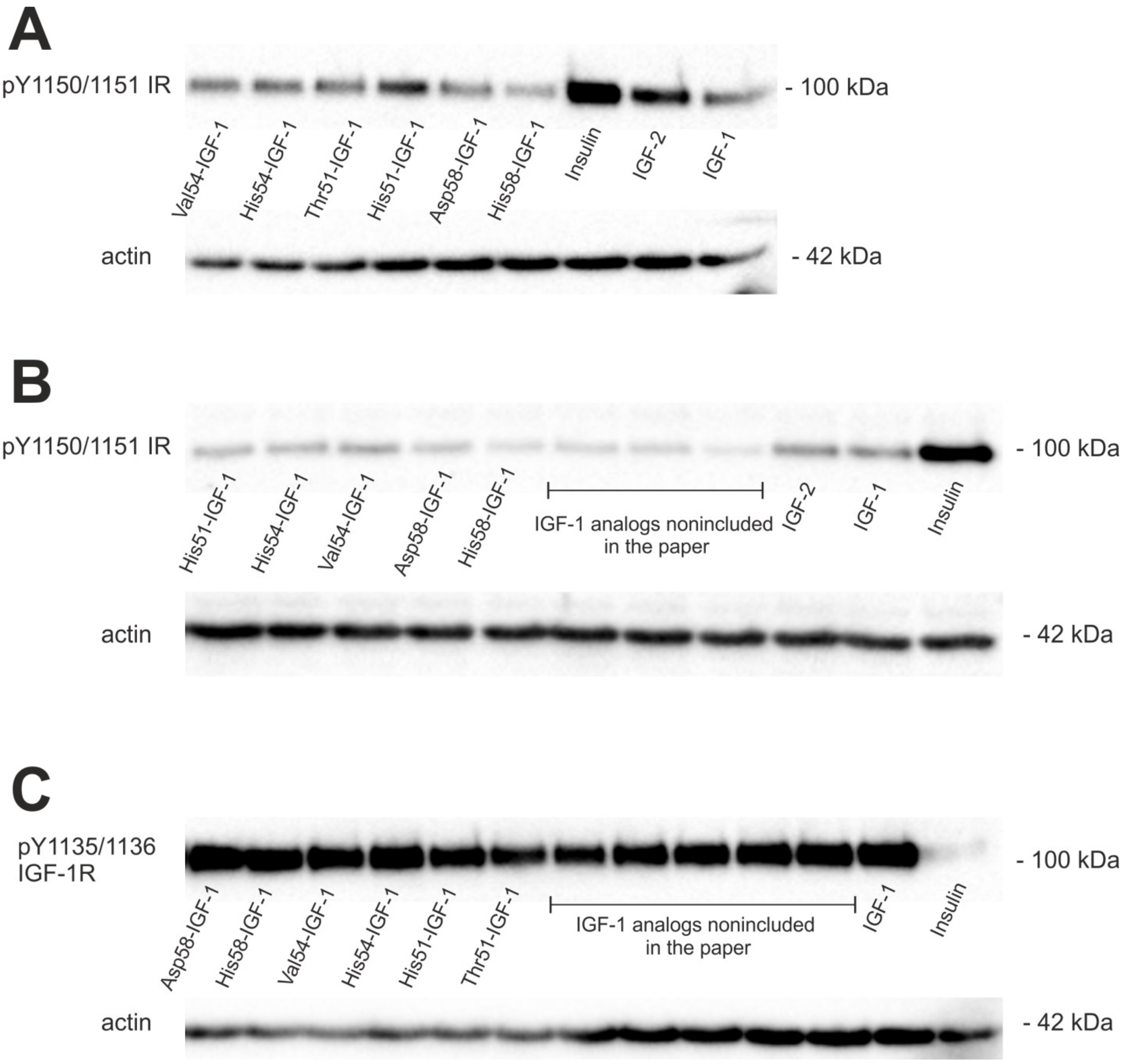

Figure S4. Representative Western blots for relative abilities of IGF-1 analogs to stimulate receptors' phosphorylation. A. IR-A transfected fibroblasts. **B.** IR-B transfected fibroblasts. **C.** IGF-1R transfected fibroblasts. Cells were stimulated with 10 nM ligands for 10 min. Membranes were cut at 75 kDa and 50 kDa standards and respective parts were developed with anti-phospho-IGF-1Rβ (Tyr1135/1136)/IRβ (Tyr1150/1151) antibody (Mr above 75 kDa) and with anti-actin antibody (Mr bellow 50 kDa). In some cases, cells were also stimulated with analogs that were not discussed in the manuscript.

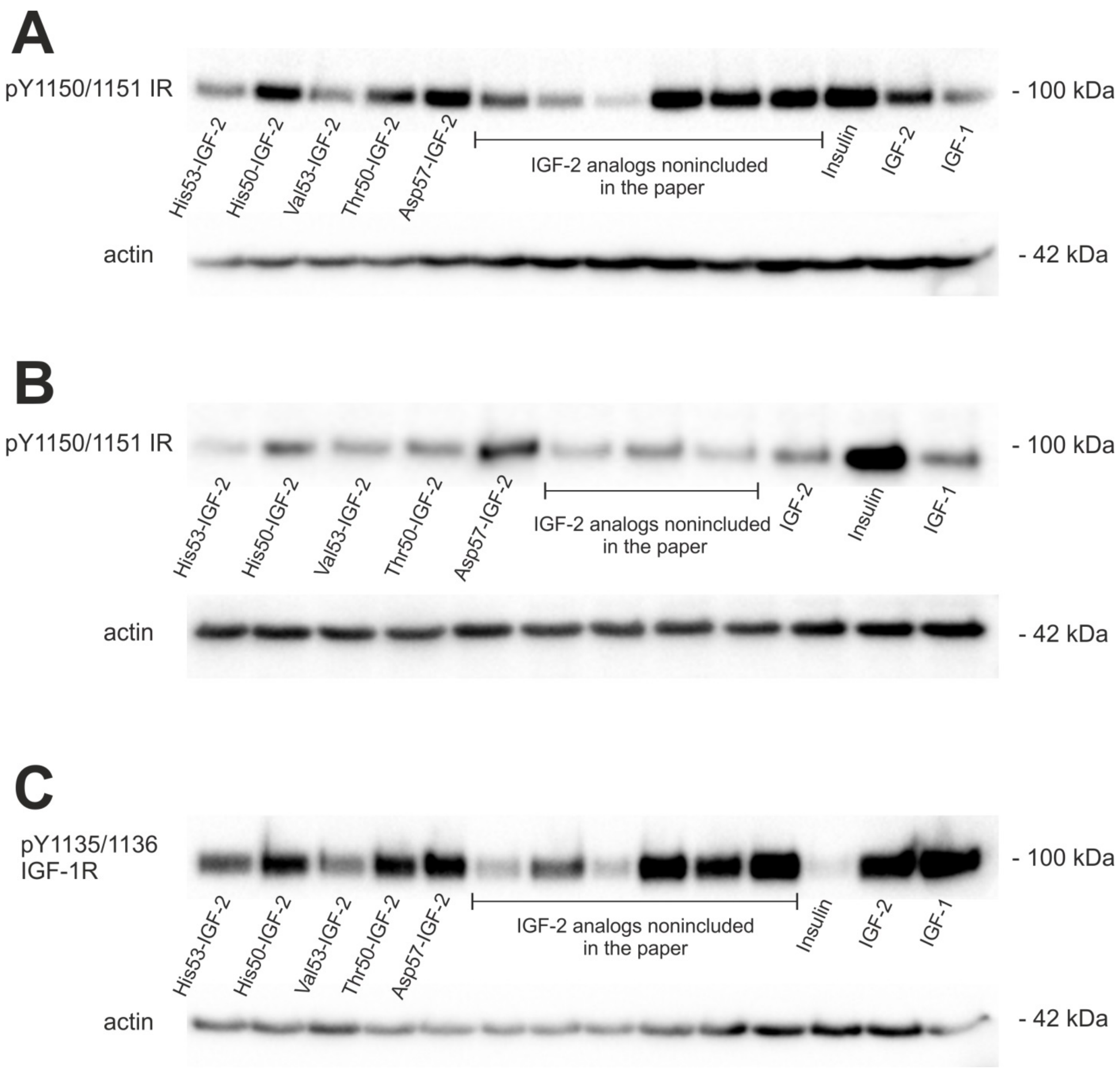

Figure S5. Representative Western blots for relative abilities of IGF-2 analogs to stimulate receptors' phosphorylation. A. IR-A transfected fibroblasts. **B.** IR-B transfected fibroblasts. **C.** IGF-1R transfected fibroblasts. Cells were stimulated with 10 nM ligands for 10 min. Membranes were cut at 75 kDa and 50 kDa standards and respective parts were developed with anti-phospho-IGF-1Rβ (Tyr1135/1136)/IRβ (Tyr1150/1151) antibody (Mr above 75 kDa) and with anti-actin antibody (Mr bellow 50 kDa). In some cases, cells were also stimulated with analogs that were not discussed in the manuscript.

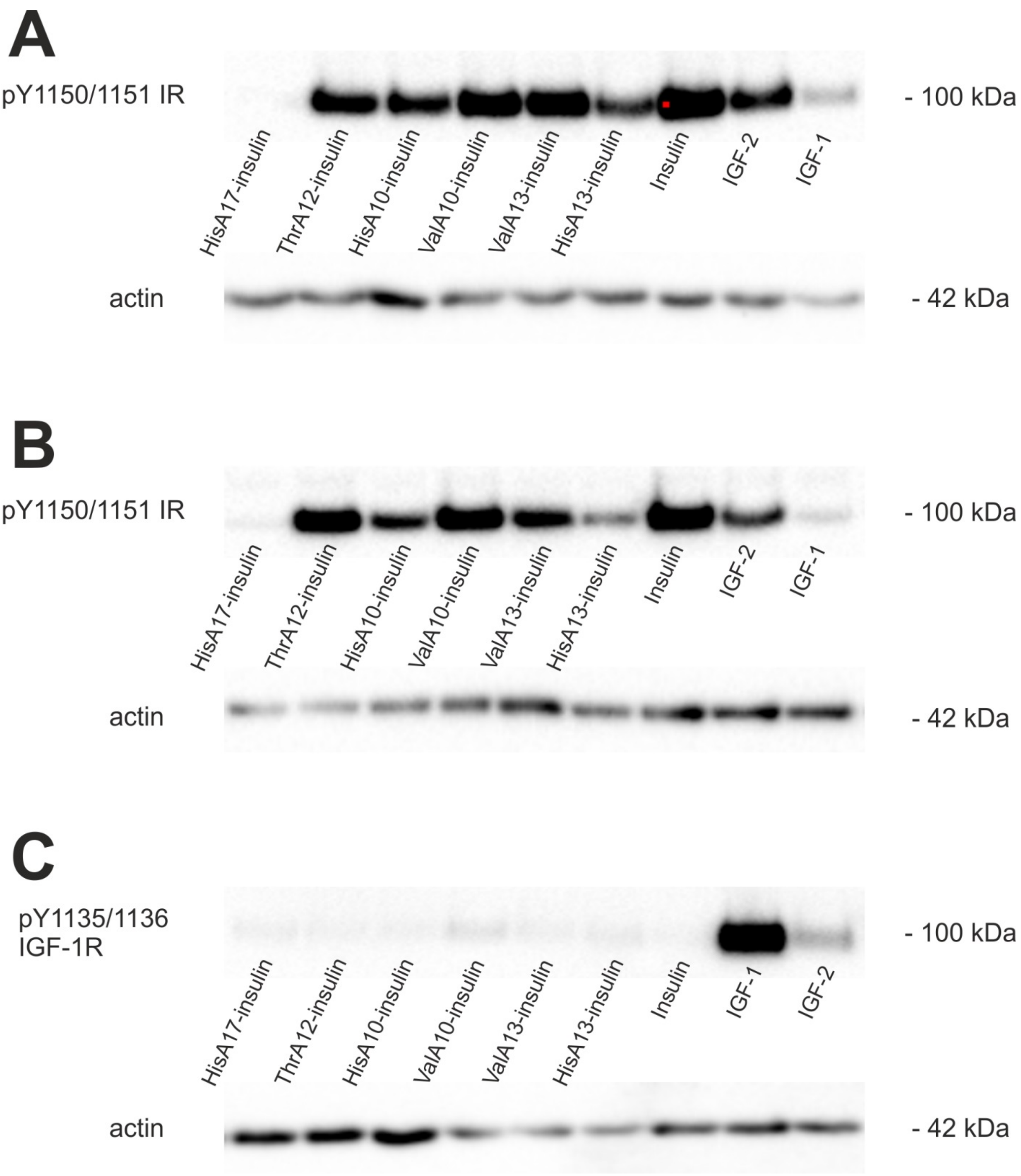

Figure S6. Representative Western blots for relative abilities of insulin analogs to stimulate receptors' phosphorylation. A. IR-A transfected fibroblasts. **B.** IR-B transfected fibroblasts. **C.** IGF-1R transfected fibroblasts. Cells were stimulated with 10 nM ligands for 10 min. Membranes were cut at 75 kDa and 50 kDa standards and respective parts were developed with anti-phospho-IGF-1Rβ (Tyr1135/1136)/IRβ (Tyr1150/1151) antibody (Mr above 75 kDa) and with anti-actin antibody (Mr bellow 50 kDa).

Table S4. Relative binding affinities of insulin analogs mutated at positions A10/A12/A3/A17, IGF-1 analogs mutated at 51/53/54/58 positions and IGF-2 analogs mutated at 52/53/57 sites. We have not found any available data on IGF-2 mutated at the position 50. Relative binding affinity is expressed in % of binding affinity of the native hormone.

Position in the native hormone	Mutation	Binding affinity (in % of the native hormone) for			References
		IR-A	IR-B	IGF-1R	
IleA10-insulin	Ser	20			Ref. (1)
Ser51-IGF-1	Ile	61			Ref. (1)
SerA12-insulin	Ala	108 (soluble receptor)			Ref. (2)
		36 (cell receptor)			A.-M. Jensen thesis referenced in Ref. (3)
Asp53-IGF-1	Ala			66	Ref. (4)
Asp52-IGF-2	Ala	133	134	87	Ref. (5)
	Asn			170	Ref. (6)
	Lys			60	Ref. (6)
	Glu			30	Ref. (6)
LeuA13-insulin	Ala	30			Ref. (2)
	Glu	20		163	Ref. (7)
Leu54-IGF-1	Ala			24	Ref. (4)
Leu53-IGF-2	Ala	25	43	40	Ref. (5)
				50	Ref. (6)
GluA17-insulin	Ala	56 (soluble receptor)			Ref. (2)
		35 (cell receptor)			A.-M. Jensen thesis referenced in Ref. (3)
Glu58-IGF-1	Ala			15	Ref. (4)
Glu57-IGF-2	Ala	17	17	34	Ref. (5)

Table S5. Chemical shifts of amino acid residues in Asp58-IGF-1 and native IGF-1. (The ^{15}N signals of prolines were not detected).

Asp58-IGF-1								native IGF-1							
	NH	^{15}N	Hα		NH	^{15}N	Hα		NH	^{15}N	Hα		NH	^{15}N	Hα
Gly1	8.42	108.82	4.17,4.19	Arg36	8.13	122.14	4.33	Gly1	8.42	108.83	4.16,4.21	Arg36	8.13	122.18	4.33
Pro2	-	*	4.42	Arg37	8.13	121.29	4.33	Pro2	-	*	4.41	Arg37	8.12	121.22	4.32
Glu3	8.57	120.09	4.47	Ala38	8.13	126.00	4.57	Glu3	8.57	120.08	4.48	Ala38	8.11	126.00	4.57
Thr4	8.00	114.44	4.50	Pro39	-	*	4.41	Thr4	8.00	114.65	4.49	Pro39	-	*	4.41
Leu5	8.24	124.29	4.44	Gln40	8.42	119.90	4.37	Leu5	8.27	124.43	4.45	Gln40	8.42	119.96	4.38
Cys6	8.18	120.48	4.68	Thr41	8.03	114.07	4.34	Cys6	8.24	120.28	4.69	Thr41	8.03	114.12	4.36
Gly7	8.65	110.54	3.80,3.93	Gly42	8.45	110.83	4.08,4.06	Gly7	8.71	110.82	3.92,3.76	Gly42	8.47	110.98	4.07,4.13
Ala8	8.57	126.73	4.09	Ile43	7.85	120.24	4.00	Ala8	8.62	127.2	4.07	Ile43	7.84	120.40	3.96
Glu9	8.09	115.84	4.14	Val44	7.89	122.07	3.73	Glu9	8.05	115.7	4.12	Val44	7.88	122.22	3.70
Leu10	7.39	121.37	4.03	Asp45	7.98	120.61	4.51	Leu10	7.33	121.32	3.99	Asp45	7.92	120.49	4.50
Val11	7.50	117.90	3.40	Glu46	7.99	118.50	4.16	Val11	7.41	117.89	3.33	Glu46	7.95	118.38	4.14
Asp12	8.09	118.14	4.41	Cys47	8.22	114.34	4.81	Asp12	8.07	117.93	4.39	Cys47	8.23	113.87	4.86
Ala13	7.75	122.95	4.22	Cys48	7.98	116.85	4.55	Ala13	7.75	123.02	4.23	Cys48	7.95	116.82	4.55
Leu14	8.10	118.70	3.87	Phe49	7.84	116.68	4.67	Leu14	8.12	118.72	3.82	Phe49	7.84	116.58	4.67
Gln15	8.13	118.45	4.12	Arg50	7.78	118.58	4.44	Gln15	8.13	118.58	4.12	Arg50	7.74	118.57	4.46
Phe16	7.69	118.69	4.44	Ser51	7.88	112.78	4.47	Phe16	7.66	118.65	4.42	Ser51	7.88	112.73	4.47
Val17	8.29	118.22	3.74	Cys52	8.83	121.20	4.83	Val17	8.42	118.02	3.69	Cys52	8.93	121.39	4.86
Cys18	8.54	116.08	4.79	Asp53	8.28	121.77	4.69	Cys18	8.59	115.77	4.80	Asp53	8.16	121.80	4.69
Gly19	7.81	109.31	3.95,3.98	Leu54	8.42	124.70	4.02	Gly19	7.73	109.2	3.96,3.99	Leu54	8.39	123.05	3.99
Asp20	8.68	122.51	4.55	Arg55	8.13	117.08	4.12	Asp20	8.74	122.73	4.54	Arg55	8.06	116.72	4.03
Arg21	8.07	118.63	4.19	Arg56	7.81	118.05	4.34	Arg21	8.05	118.5	4.17	Arg56	7.82	118.63	4.20
Gly22	7.55	104.57	3.77,3.98	Leu57	7.80	119.18	4.20	Gly22	7.46	104.24	4.01,3.77	Leu57	7.86	118.53	4.15
Phe23	7.58	115.20	5.01	**Asp58**	8.05	115.18	4.56	Phe23	7.54	114.6	5.07	**Gln58**	7.87	114.85	4.22
Tyr24	8.44	119.68	4.73	Met59	7.67	117.74	4.24	Tyr24	8.51	119.54	4.70	Met59	7.57	117.18	4.21
Phe25	8.19	118.04	4.67	Tyr60	7.93	117.65	4.56	Phe25	8.17	118.03	4.69	Tyr60	7.90	117.19	4.52
Asn26	8.12	119.61	4.73	Cys61	7.46	116.20	5.02	Asn26	8.12	119.5	4.77	Cys61	7.41	115.92	5.04
Lys27	8.11	122.14	4.47	Ala62	8.24	126.53	4.43	Lys27	8.15	122.23	4.48	Ala62	8.26	126.77	4.42
Pro28	-	*	4.47	Pro63	-	*	4.40	Pro28	-	*	4.46	Pro63	-	*	4.40
Thr29	8.11	113.54	4.31	Leu64	8.18	121.97	4.23	Thr29	8.11	113.62	4.31	Leu64	8.20	122.05	4.22
Gly30	8.23	110.34	3.89,3.95	Lys65	8.24	123.48	4.61	Gly30	8.23	110.36	3.95,3.89	Lys65	8.26	123.65	4.60
Tyr31	8.07	120.06	4.55	Pro66	-	*	4.38	Tyr31	8.07	120.08	4.56	Pro66	-	*	4.38
Gly32	8.34	110.86	3.97,3.88	Ala67	8.31	124.65	4.29	Gly32	8.35	110.92	3.97,3.88	Ala67	8.31	124.72	4.29
Ser33	8.18	115.69	4.47	Lys68	8.18	120.10	4.35	Ser33	8.19	115.76	4.47	Lys68	8.18	120.17	4.35
Ser34	8.34	117.25	4.49	Ser69	8.26	117.74	4.44	Ser34	8.35	117.32	4.49	Ser69	8.26	117.79	4.43
Ser35	8.16	117.07	4.44	Ala70	8.02	129.97	-	Ser35	8.16	117.12	4.44	Ala70	8.04	129.83	4.20

Table S6. Chemical shift differences between Asp51-IGF-1 and native IGF-1.

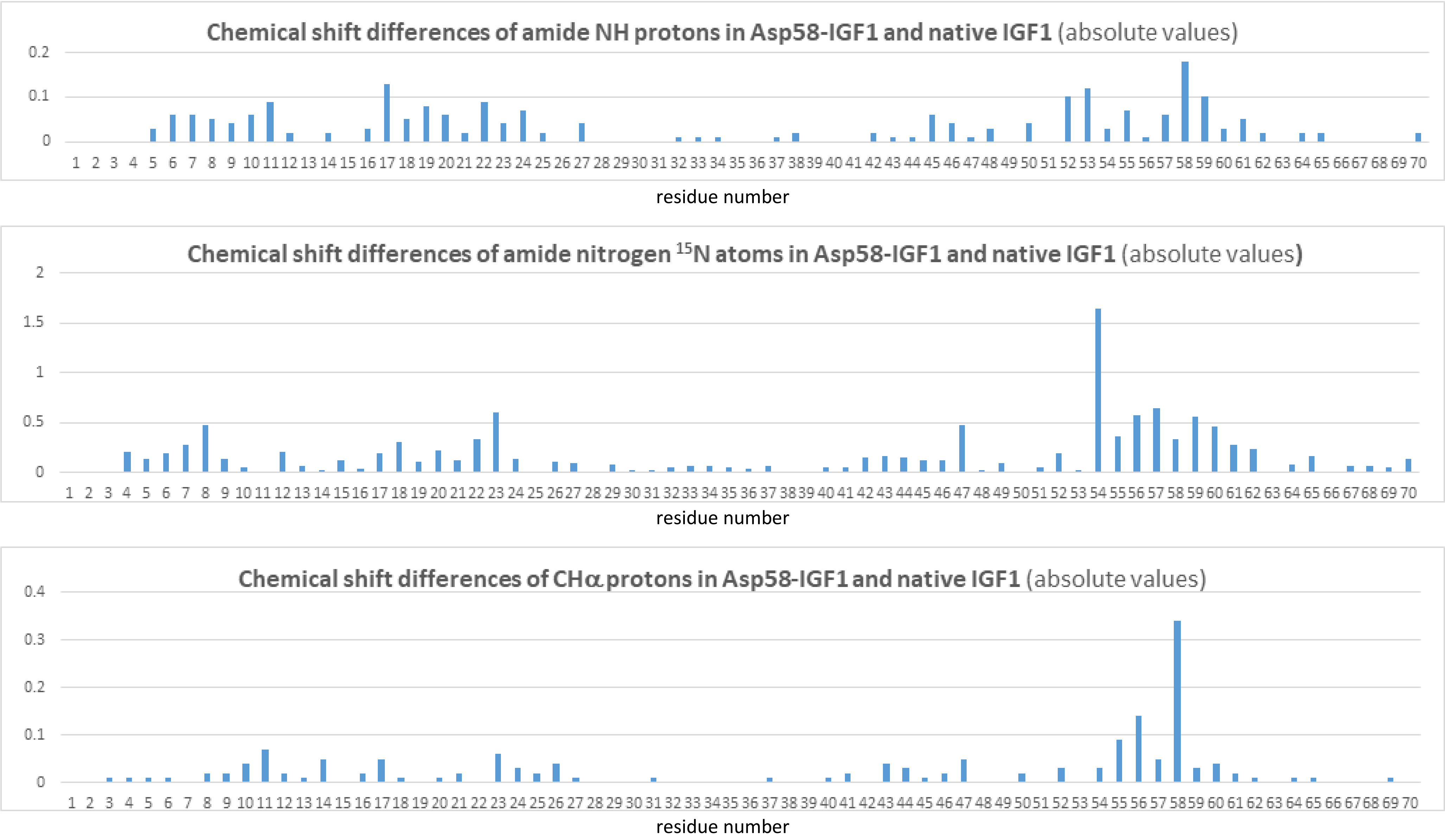

Table S7. NOEs statistics

Distance restraints (NOEs)	
Intra-residue	330
Sequential (|*i*-*j*| = 1)	159
Medium range (|*i*-*j*|≤ 4)	48
Long-range (|*i*-*j*| ≥ 5)	57
Ambiguous	147
Total	741
Dihedral angle restraints	
ϕ	18
Ψ	18
χ	3
Total	39
J-coupling restraints	
Total	10
*Ensemble statistics**	
Backbone (Å)	0.46
Heavy atoms (Å)	0.92
All atom (Å)	1.09

----

*only well-defined parts of the structure: Ala8-Cys18, Ile43-Tyr60

Differential Contact Maps calculated from metadynamics of the insulin B-chain C-terminus opening

The inter-residue contacts with a negative difference in contact lifetime between the wild-type and mutants are those that are more frequently present in mutant compared to wild-type insulin (colored in orange in **Figure S7**). A range of hydrophobic contacts between the B-chain N-terminus (PheB1, ValB2) and the A chain (LeuA13, LeuA16) were established to maintain the collapsed state for HisA10 and HisA13 mutants, as well as the closed state for the HisA17 mutant. Contacts between the B-chain N-terminus and residues in the B-chain α-helix were more frequently present in the collapsed/closed states of the His-insulin mutants. For example, the contacts in HisA13 mutant between residues HisB5-GlyB8, GlnB4-HisB10, GlnB4-GluB13 contributed to the partial collapse of the B-chain α-helix. For the native-like affinity ThrA12 contacts that were formed more frequently than in the wild type (for example IleA2, ValA3 with LeuB15, LeuB11) maintained the hydrophobic interface between A/B chains in a more compact state.

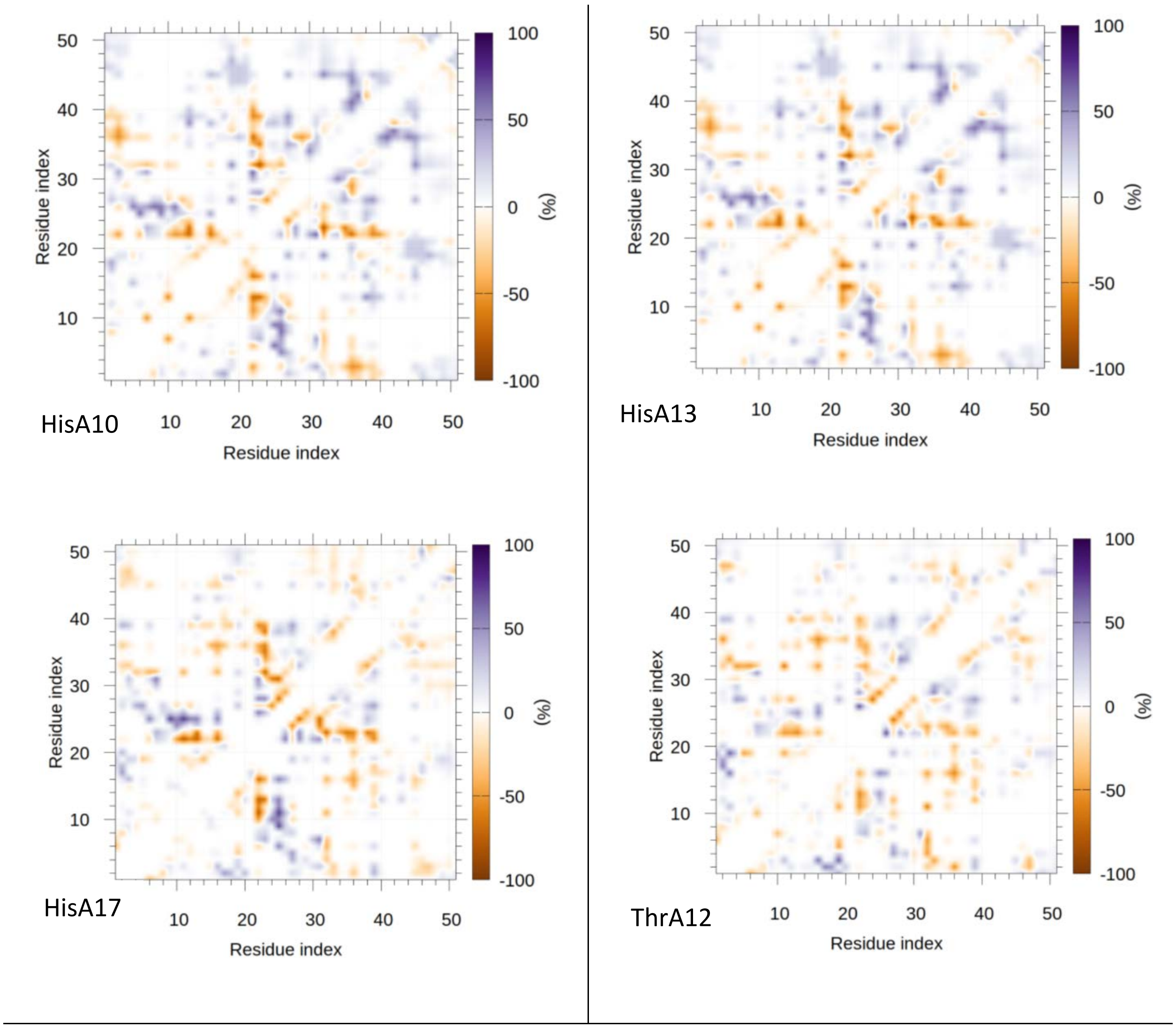

Figure S7. Change in total interaction time for insulin mutants calculated as the difference in fraction of time contacts are present between residues for the wild type and the mutant (a negative difference signifies a contact less frequently present in wild type or a contact gained in mutant). The residue index goes from 1-21 for A chain and 22-51 for B chain of insulin. HisA10-insulin, Left upper panel; HisA13-insulin, right upper panel; HisA17-insulin, left lower panel; ThrA12-insulin, right lower panel.

Structural characterization of the metadynamics ensembles

The insulin hydrophobic core comprises residues IleA2, ValA3, GlyB8, LeuB11, ValB12, LeuB15, PheB24 and TyrB26 (8). We calculated its size in terms of the radius of gyration R_{gyr} comprising all atoms for the respective residues. The hydrophobic core is more compact for the mutants compared to the wild-type insulin, except for the HisA10-mutant which collapses to extended states (**Figure S8A**). Connected to the extent of the hydrophobic collapse, we observed different protein-water hydrogen bonding local to the point of mutation. The number of water-protein hydrogen bonds was determined for a shell of the radius 1 nm around the Cα (CA) atom of a mutated residue. With OH and NH groups regarded as donors and O and N atoms as acceptors, the donor-acceptor cutoff distance was set to 0.35 nm and the angle hydrogen-donor-acceptor to 30°. The number of water-protein H-bonds in a sphere of a 1 nm radius around the Cα atom of a mutated/wild-type residue is plotted in **Figure S8B** comparing the HisA17-mutant and ThrA12-mutant in red to wild-type native insulin in black. The dynamics of H-bond formation is more stable in the closed compact state of HisA17-mutant. On the other hand, the bulkier ThrA12 is buried inside the core and forms less H-bonds with water than the wild-type SerA12.

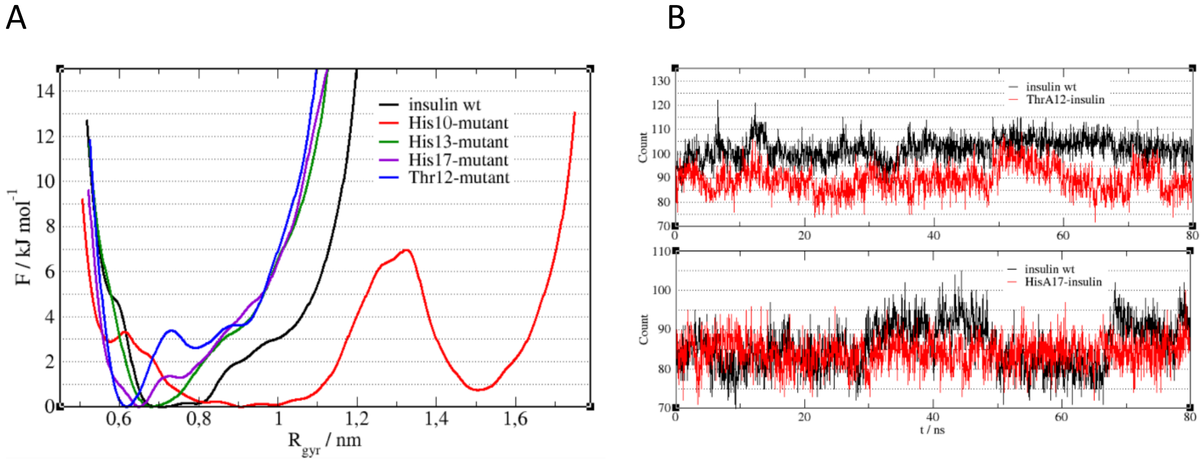

Figure S8. A. Free energy profiles with respect to the radius of gyration of residues defining the hydrophobic core. **B.** The number of protein-solvent hydrogen bonds in the sphere of 1 nm around Cα atoms of mutated residues: Cα (ThrA12) in ThrA12-insulin mutant and Cα (SerA12) in wild-type insulin (up), Cα (HisA17) in HisA17-insulin mutant and Cα (GluA17) in native insulin (down). Insulin wt is a native wild-type insulin.

References

1. Gauguin, L., Klaproth, B., Sajid, W., Andersen, A. S., Mcneil, K. A., Forbes, B. E., and De Meyts, P. (2008) Structural basis for the lower affinity of the insulin-like growth factors for the insulin receptor. *J. Biol. Chem.* **283**, 2604-2613

2. Kristensen, C., Kjeldsen, T., Wiberg, F. C., Schaffer, L., Hach, M., Havelund, S., Bass, J., Steiner, D. F., and Andersen, A. S. (1997) Alanine scanning mutagenesis of insulin. *J. Biol. Chem.* **272**, 12978-12983

3. De Meyts, P. (2015) Insulin/receptor binding: The last piece of the puzzle? *Bioessays* **37**, 389-397

4. Gauguin, L., Delaine, C., Alvino, C. L., McNeil, K. A., Wallace, J. C., Forbes, B. E., and De Meyts, P. (2008) Alanine scanning of a putative receptor binding surface of insulin-like growth factor-I. *J. Biol. Chem.* **283**, 20821-20829

5. Alvino, C. L., McNeil, K. A., Ong, S. C., Delaine, C., Booker, G. W., Wallace, J. C., Whittaker, J., and Forbes, B. E. (2009) A Novel approach to identify two distinct receptor binding surfaces of insulin-like growth factor II. *J. Biol. Chem.* **284**, 7656-7664

6. Delaine, C., Alvino, C. L., McNeil, K. A., Mulhern, T. D., Gauguin, L., De Meyts, P., Jones, E. Y., Brown, J., Wallace, J. C., and Forbes, B. E. (2007) A novel binding site for the human insulin-like growth factor-II (IGF-II)/mannose 6-phosphate receptor on IGF-II. *J. Biol. Chem.* **282**, 18886-18894

7. Schaffer, L. (1994) A model for insulin binding to the insulin receptor. *Eur. J. Biochem.* **221**, 1127-1132

8. Papaioannou, A., Kuyucak, S., and Kuncic, Z. (2015) Molecular dynamics simulations of insulin: elucidating the conformational changes that enable its binding. *Plos One* **10**
